# Potential of Nanoparticle‐Based Phototherapies for Future Treatment of Uveal Melanoma

**DOI:** 10.1002/advs.77396

**Published:** 2026-08-31

**Authors:** Emilie Lambert, Christophe Nguyen, Sean Edward Dunn, Magali Gary‐Bobo, Jérôme Lacombe, Justin Moser, Frédéric Zenhausern, Jean‐Olivier Durand, Frédérique Cunin

**Affiliations:** ^1^ ICGM CNRS ENSCM University of Montpellier Montpellier France; ^2^ IBMM CNRS ENSCM University of Montpellier Montpellier France; ^3^ Center for Applied Nanobioscience and Medicine & Department of Basic Medical Sciences College of Medicine Phoenix University of Arizona Phoenix Arizona USA; ^4^ HonorHealth Research Institute Scottsdale Arizona USA

**Keywords:** laser therapy, nanomedicine, nanoparticles, uveal melanoma

## Abstract

This review aims at exploring nanoparticle‐based phototherapies of uveal melanoma (UM), the most common primary intraocular malignancy in adults with an estimated incidence of 5–7 cases per million individuals per year, a disproportionately high metastatic risk and poor prognosis in advanced stages. UM exhibits unique biological and anatomical features, including an immune‐privileged ocular microenvironment, the blood–ocular barrier, and high melanin content, contributing to therapeutic complexity. Current treatments, including radiotherapy, surgery, and systemic therapies, achieve local tumor control but remain limited by adverse effects, recurrence, and insufficient efficacy against metastatic disease. Laser‐based therapies, including transpupillary thermotherapy and photodynamic therapy, offer minimally invasive alternatives. However, their performance is constrained by limited light penetration, melanin‐induced uneven absorption, and incomplete tumor eradication. Recent advances in nanotechnology offer opportunities to overcome these limitations. Nanoparticle‐based phototherapies can improve tumor targeting, photosensitizer delivery, and localized activation while reducing off‐target effects. Multifunctional nanoplatforms may combine imaging, drug delivery, and synergistic therapies such as immunotherapy or chemotherapy. Although promising preclinical results highlight improved tumor control and antitumor responses, challenges related to safety, biodistribution, and clinical translation remain. This review discusses these challenges and future directions for optimized nanoplatforms to improve disease management and metastasis prevention.

## Graphical abstract

This review evaluates nanoparticle‐based phototherapies for uveal melanoma, highlighting emerging strategies to enhance tumor targeting, light delivery, and treatment precision. Preclinical data indicate improved efficacy and reduced toxicity, supporting their potential to enhance localized treatment and future translational advances.

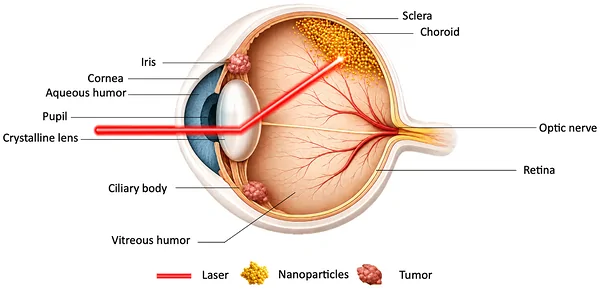

## 1 Introduction

**FIGURE 1:**
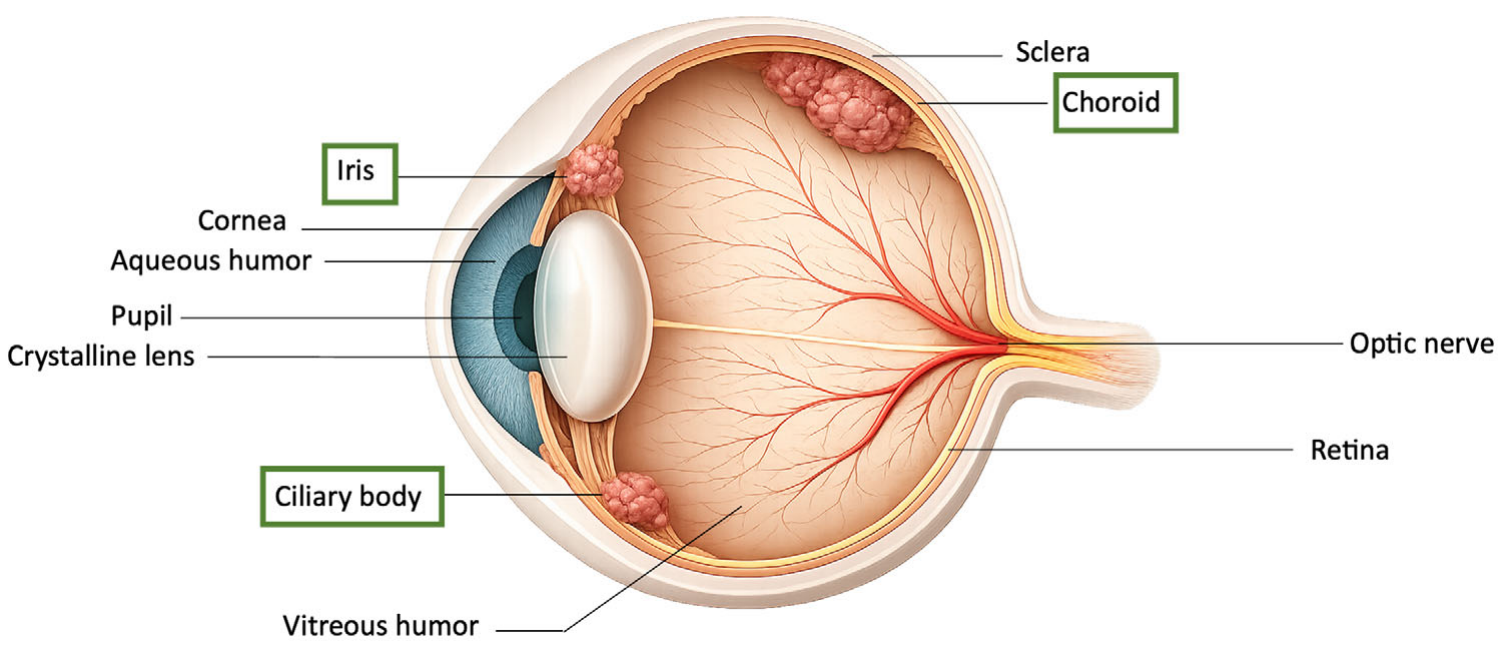
Anatomical representation of the human eye showing uveal UM tumor locations (generated by the authors by mean of AI, Illustrae).

Uveal melanoma (UM) is the most common malignant intraocular tumor in adults and accounts for approximately 3%–5% of all melanoma cases. Although considered a rare malignancy, UM remains a major clinical concern because of its high metastatic potential and poor prognosis following dissemination. The tumor arises predominantly from melanocytes located within the choroid (85%–90%), followed by the ciliary body (5%–8%) and iris (3%–5%), structures that together constitute the uvea, the middle vascular layer of the eye (Figure 1) [1, 2, 3, 4]_._ Epidemiological studies indicate that UM is most frequently diagnosed in older adults, with a median age at diagnosis of approximately 62 years, and suggest that genetic predisposition and pigmentation‐related factors (fair skin, light‐colored eyes, inability to tan, ocular or oculodermal melanocytosis, uveal nevi, and germline BAP1 mutations) play important roles in disease development [2, 3, 4, 5]. Although advances in diagnostic methods and therapeutic interventions have been achieved over recent decades, the incidence of UM has remained relatively stable [6, 7, 8, 9]. Often asymptomatic in its early stages, UM may manifest with blurred vision, photopsia (flashes), floaters, or vision impairment as tumor growth progresses. It has a strong propensity for metastatic dissemination, particularly to the liver, and less frequently to the lungs [10]. Prognosis is usually poor, with a median survival is only 4–6 months after metastasis. Clinical outcomes depend on factors including tumor size, anatomical location, and molecular alterations, with monosomy 3 and *BAP1* mutations representing major adverse prognostic indicators [11, 12, 13]. Diagnosis of UM is established through ophthalmoscopy, ultrasonography, or optical coherence tomography (OCT) [1, 14, 15]. Beyond its clinical aggressiveness, UM represents a biologically distinct intraocular malignancy whose pathophysiology is strongly influenced by the ocular immune‐privileged environment. This specialized milieu is maintained through local immunoregulatory mechanisms involving high concentrations of immunosuppressive soluble factors, presence of immunosuppressive cell membrane molecules, reduced major histocompatibility complex expression, and anterior chamber‐associated immune deviation [16], collectively limiting effector immune responses and promoting tumor immune escape. In addition, the blood–ocular barrier (BOB), formed by retinal vascular endothelial tight junctions and retinal pigment epithelial cells, restricts the diffusion of immune cells and systemically administered therapeutic agents into intraocular compartments, thereby reducing intratumoral drug bioavailability and contributing to therapeutic resistance.

Another important characteristic of UM is its frequently elevated melanin content. Melanin exhibits strong wavelength‐dependent optical absorption, particularly in the visible and near‐infrared (NIR) spectrum, resulting in uneven energy distribution within tumor tissues. During laser‐mediated interventions, such optical heterogeneity may generate localized thermal gradients and high‐energy‐density regions, potentially causing collateral damage to adjacent structures including the retina, choroid, and optic nerve tissues [17]. Taken together, these distinctive characteristics of UM emphasize the need for therapies specifically adapted to its unique biological and clinical features.

Current treatments for UM include surgical resection, enucleation for large or painful tumors (with severe psychological consequences), radiotherapy, particularly brachytherapy, and laser therapies, as local therapies for the management of primary tumors. Liver‐directed therapies or systemic immunotherapy are generally employed for patients with metastatic spread, particularly with hepatic involvement [18, 19, 20].

The human eye is an optically transparent organ which allows efficient transmission of visible light (≈400–700 nm) to the retina. This is enabled by specialized ocular structures (the cornea, crystalline lens, aqueous humor, and vitreous humor), which exhibit high ordered architectures and minimal light‐scattering properties [21, 22, 23]. Light transmission in the eye is wavelength‐dependent, with ultraviolet (UV) radiation mainly absorbed by the cornea and lens, whereas melanin present in the iris, retinal pigment epithelium, choroid, and ciliary body absorbs excess of light, limits excessive intraocular light scattering and phototoxicity, while optimizing visual functions [24, 25]. The eye is a logical well‐suited organ for laser‐based therapies, because both transparency and selective absorption enable precise and controlled light delivery to intraocular tissues and tumors, while minimizing damage to surrounding healthy structures [26, 27, 28]. Laser applications have been established in ophthalmology since the 1960s and became widely adopted in clinical practice from the 1970s onward, supporting numerous diagnostic and therapeutic procedures, including UM management [29].

Laser‐based approaches for UM include transpupillary thermotherapy (TTT) and photodynamic therapy (PDT) [18, 30]. TTT utilizes infrared laser irradiation delivered through the pupil to induce cellular necrosis of pigmented tumors, and is generally indicated for small choroidal melanomas or as an adjunct to radiotherapy [31, 32]. PDT is a minimally invasive and selective therapy based on the combined action of light and photosensitizing agents to induce localized cytotoxic effects within tumor cells [33, 34, 35].

Despite the availability of multiple therapeutic options for UM, important clinical and biological limitations persist. Local treatments, including surgery, radiotherapy, and laser‐based approaches, remain constrained by variables such as tumor size, location, thickness, depth, pigmentation, and the risk of functional complications, such as visual impairment or retinal detachment [34, 35, 36, 37, 38, 39]. Furthermore, local recurrence and metastases progression may occur even after successful primary tumor treatment [37, 40, 41, 42, 43]. Within the current therapeutic landscape of UM, this review aims at focusing on laser and photo‐induced therapeutic strategies, intended for primary tumor treatment, potentially accessible by light, and discusses their recent advances particularly in combination with nanomaterials, which may offer new opportunities in this challenging field. The review critically examines their mechanisms of action, current preclinical and clinical evidence, safety considerations, and translational potential.

## 2 Current Standard Treatments for UM

UM is a malignancy in which primary tumors develop within the uveal tract of the eye. Despite local treatment management, UM carries a high risk of metastasis. Because the uveal tract lacks conventional lymphatic drainage, metastatic dissemination occurs predominantly through the hematogenous route. The liver is the dominant metastatic site (89%), followed by the lungs (29%), bones (17%), and brain (9%) [2, 44]. Various therapeutic strategies are used for the management of both primary and metastatic UM (mUM) [18, 45, 46].

### 2.1 Management of mUM

mUM remains a therapeutic challenge due to its tendency to spread rapidly, particularly to the liver, and its resistance to existing clinical treatments [10, 20]. Several therapeutic approaches are being explored or employed and can be summarized into three main categories. First, conventional chemotherapy using alkylating agents, although traditionally applied in the treatment of various cancers, demonstrates limited efficacy against metastases (approximately 10%) and is highly toxic to patients [47, 48]. Second, systemic treatments include (i) immunotherapy, with tebentafusp (TEBE, commercially known as Kimmtrak), a bispecific ImmTAC (Immune mobilizing monoclonal T‐cell receptors Against Cancer) targeting gp100 and CD3. It represents the first systemic therapy to demonstrate a meaningful overall survival benefit in mUM, although its use is limited to HLA‐A*02:01‐positive patients [49, 50, 51], and has immune‐related toxicities that require monitoring [52]; immune checkpoint inhibitors (ICIs) can also be used (anti‐PD‐1 or anti‐CTLA‐4) [53, 54], and (ii) targeted therapies using enzyme‐inhibiting molecules, such as selumetinib, which inhibits the MEK enzyme. These approaches are designed to specifically target tumor cells and their signaling pathways to improve therapeutic outcomes [55, 56]. Third, given the predominance of hepatic metastases, several locoregional strategies directly targeting the liver are employed. The main approaches include percutaneous hepatic perfusion, partial hepatectomy, transarterial chemoembolization (TACE), radiofrequency ablation, and embolization [57, 58, 59, 60]. Beyond this, research is focusing on innovative combination approaches, such as combining standard immunotherapies with radiotherapy or with targeting systems to enhance immune response. Other emerging strategies include testing newer ICIs like anti‐LAG‐3 or anti‐TIGIT, and developing treatments that modify the tumor microenvironment (TME) to make cancer cells more visible and responsive to the immune system [54, 61, 62, 63].

### 2.2 Standard Local Treatments for Primary UM

The treatment of primary UM tumors is primarily based on local therapies, applied directly to the tumor while aiming to achieve optimal tumor control and preserve patients’ visual acuity, particularly by protecting ocular tissues whenever possible. Radiotherapy is considered the clinical standard treatment for UM (local disease) [37, 64]. It encompasses several techniques, which include charged particle therapy (CPT) [66, 67] such as proton beam therapy (PBT) [63, 64, 65], and brachytherapy, the most well‐known and widely used technique [65, 66, 67]. CPT is a form of external beam radiotherapy, better suited for large tumors or those located near the optic nerve, whereas brachytherapy is an internal radiotherapy indicated for small to medium‐sized tumors. Overall, all approaches aim to provide high local tumor control while minimizing ocular damage based on the specific characteristics of the tumors [30, 68]. Surgical intervention, such as local excision or enucleation, represents an additional option, although it is less preferred (as patients lose vision), and generally indicated for large tumors [38, 45, 69]. These therapeutic approaches have been intensively documented and reviewed and will not be detailed in the present review.

Despite their effectiveness, radiotherapy and surgery are associated with several limitations that can restrict their use or therapeutic success. Radiotherapy, whether external or internal, inevitably exposes surrounding ocular structures to radiation doses that can cause significant side effects, such as radiation retinopathy, maculopathy, secondary glaucoma, or optic neuropathy. The likelihood of such complications increases for large or posteriorly located tumors, for tumors near the fovea or optic nerve, or when higher radiation doses are necessary to achieve effective tumor control [70, 71, 72, 73]. Furthermore, specific tumor locations and morphologies may compromise irradiation precision, reducing radiotherapy suitability and effectiveness and potentially leading to tumor recurrence, which has been reported in 20% of cases [74, 75]. Surgical interventions remain more invasive and are associated with significant perioperative and postoperative risks, including hemorrhage, retinal detachment, or, in the case of enucleation, complete loss of the eye both functionally and anatomically, with associated psychological impact. In addition, there is a potential risk of tumor dissemination [69]. Overall, local tumor control appears not to reliably prevent liver metastases. These limitations have encouraged the development of less invasive therapeutic approaches that can more specifically target lesions while sparing healthy tissues. In this regard, laser therapies and photo‐induced techniques, such as TTT and PDT, have emerged as promising alternative treatments. They are minimally invasive, not requiring surgery nor extensive recovery time, with minimal side effects (Tables 1 and 2).

**TABLE 1:** Summary of the current standard treatment options for UM.

Therapy category	Therapy type	Main use	Advantages	Limitations	Unmet need	Refs.
**Systemic therapies for mUM**	Conventional chemotherapy	Systemic administration of cytotoxic alkylating agents (dacarbazine, temozolomide, etc.)	Widely available, simple administration, established used in clinical practice	Very low response rates (∼10%), chemoresistance, significant toxicity, limited survival benefit	Effective systemic therapies with durable responses	[47, 48]
	Immunotherapy or ICIs therapy	Enhances antitumor immunity through gp100‐targeted T‐cell redirection (TEBE) or PD‐1/CTLA‐4 immune checkpoint inhibition, restoring T‐cell function	TEBE: first systemic therapy to demonstrate significant overall survival benefit in mUM, current standard first‐line treatment for eligible patients ICIs: potential for durable responses in a subset of patients; dual ICI generally superior to monotherapy	**TEBE**: restricted to HLA‐A*02:01‐positive patients; immune‐related adverse events (rash, pyrexia, hypotension, pruritus) **ICIs**: low response rates due to an immune‐evasive TME and low mutational burden; increased toxicity with dual ICI therapy	Development of effective immunotherapies for HLA‐A*02:01‐negative patients, identification of predictive biomarkers, strategies to overcome immune resistance and improve response durability	[49, 50, 51, 52, 53, 54]
	Enzyme‐targeting therapies (MEK, PKC, etc.)	Target key signaling pathways involved in UM progression (those activated downstream of GNAQ/GNA11 mutations). Representative examples: MEK inhibitors (selumetinib, other emerging pathway‐targeted agents)	Mechanism‐based approach specifically targeting molecular drivers of tumor growth; potential for combination strategies	Limited clinical efficacy, frequent resistance development, lack of demonstrated overall survival benefit in most studies	More effective molecular targets, predictive biomarkers, and durable combination regimens	[55, 56]
**Locoregional therapies for liver metastases**	Percutaneous hepatic perfusion	Delivers high‐dose chemotherapy directly to the liver, while limiting systemic exposure	Improved hepatic disease control, useful for liver‐dominant disease	Invasive procedure requiring specialized centers, recurrence remains common	Durable hepatic control and prevention of extrahepatic progression	[57, 58, 59, 60]
	Partial hepatectomy	Surgical resection of isolated/ resectable liver metastases	Potentially prolonged survival in highly selected patients	Applicable to a minority of patients; recurrence rates remain high	Better patient selection and adjuvant strategies to prevent relapse	
	Transarterial chemo‐emolization (TACE)	Intra‐arterial delivery of chemotherapy combined with embolization of tumor‐feeding vessels	Liver‐targeted therapy with reduced systemic exposure	Often temporary responses, repeated procedures frequently required	Improved durability and integration with systemic therapies	
	Radiofrequency ablation	Thermal destruction of hepatic metastases through localized heating	Minimally invasive and repeatable	Limited to small and accessible lesions; frequent recurrence	Expanded applicability and improved local control	
	Embolization	Occlusion of tumor‐feeding hepatic arteries inducing ischemic tumor necrosis	Regional disease control with limited systemic toxicity	Limited long‐term efficacy and frequent recurrence	Durable local control and combination strategies	
**Radiotherapy for primary UM**	PBT	External beam radiotherapy using proton particles, suitable for large tumors or lesions located near critical ocular structures (e.g., optic nerve)	High local tumor control, eye‐preserving approach, suitable for challenging tumor locations	Radiation retinopathy, maculopathy, optic neuropathy, glaucoma, visual deterioration, possible local recurrence	Improved irradiation precision, preservation of visual function	[76, 77, 78]
	CPT	External radiotherapy using charged particles to deliver highly conformal radiation doses, while limiting exposure of surrounding tissues	High local tumor control and favorable dose distribution	Radiation‐induced ocular complications, limited availability and recurrence risk	Reduced ocular toxicity and broader accessibility	[79, 80]
	Brachytherapy	Internal radiotherapy using episcleral radioactive plaques, mainly indicated for small‐ to medium‐sized tumors	Most widely used treatment, excellent local control, eye preservation	Radiation‐associated ocular complications and visual impairment, local recurrence remains possible	Improved tumor selectivity and reduced damage to healthy ocular structures	[65, 66, 67]
**Surgery for primary UM**	Local resection	Surgical excision of accessible tumors while preserving the ocular globe	Immediate tumor removal and histopathological evaluation	Invasive procedure associated with hemorrhage, retinal detachment, and possible tumor dissemination	Safer and less invasive tissue‐sparing approaches	[38, 45, 69]
	Enucleation	Complete removal of the eye, generally reserved for large, advanced, or non‐salvageable tumors	Definitive local tumor removal	Complete loss of the eye and vision, psychological impact, and surgical morbidity	Effective globe‐preserving alternatives for advanced disease	[38, 45, 69]

**TABLE 2:** Comparative summary of laser‐based therapeutic modalities and their limitations for primary UM.

Modality	Light or activation principle	Mechanism of action	Typical indication/ Tumor profile	Main limitations and ocular complications	Refs.
**Photocoagulation**	Direct irradiation with visible laser light (argon:488–514 nm, krypton: 647 nm, or diode lasers: 488–750 nm) Light absorption by endogenous chromophores (melanin and hemoglobin) Irradiation parameters: spot size 50–500 µm, exposure time 0.5–1 **s**, power 0.5–1 W	Photothermal effect: conversion of light into heat induces protein denaturation, vascular coagulation, and thermal necrosis of tumor cells. Limited penetration depth: 0.2–1 mm	Small UM lesions distant from the fovea and optic disc Basal diameter: 4.5–10 mm/ Thickness: 1.5–3.5 mm	Limited penetration depth: 0.2–1 mm incomplete eradication of thick lesions, difficulty confirming complete tumor destruction, recurrence risk, strong dependence on pigmentation and tumor location, retinal/vitreous hemorrhage, vascular occlusions, retinal macular edema, tumor extensions, scotomas (resulting in partial or complete visual function loss, and gradual discontinuation of photocoagulation)	[15, 81, 82, 83, 84, 85, 104, 117]
**TTT**	Direct irradiation with a NIR diode laser (810 nm), absorption mainly by melanin‐rich tumor tissues Irradiation parameters: spot size 1.5–4.5 mm, power 0.3–0.9 W, exposure time **∼**1 min	NIR light‐induced controlled photothermal hyperthermia (45–60°C): induces thermal necrosis, vascular injury, ischemia, and apoptosis	Small UM tumors located in the choroid Basal diameter **<** 2 mm**/** Thickness < 4.5 mm thickness	Limited efficacy in thicker tumors incomplete destruction of deep tumor cells, recurrence (**∼**9%–23%), difficult treatment near the fovea or optic disc, retinal injury, retinal or vitreous hemorrhage, scotomas, vascular occlusions, macular edema, retinal detachment, inflammatory reactions, and visual impairment	[31, 32, 86, 87, 88, 89, 90, 91, 92, 93, 107, 108, 109, 110]
**PDT**	Systemic administration of a photosensitizer followed by red‐light activation (630–690 nm) Preferential accumulation within tumor cells and/or tumor vasculature prior to irradiation TAP protocol: VP 6 mg/m^2^, irradiation at 600 mW/cm^2^ for 83 s (fluence 50 J/cm^2^)	Photochemical effect: photoactivation of the photosensitizer generates ROS (singlet oxygen), inducing tumor cell apoptosis/necrosis, endothelial damage, vascular occlusion, and ischemic tumor destruction	Small UM tumors, amelanotic or lightly pigmented lesions, located in the choroid Basal diameter: 3.5–8 mm**/** Thickness **≤** 3 mm	Reduced efficacy in highly pigmented tumors, limited optical penetration, dependence on photosensitizer uptake and tumor vascularization, variable responses, recurrence (16%–60%), retinal atrophy, hemorrhage, edema, vascular occlusions, and direct visual impairment (for juxtapapillary, juxtafoveal, or subfoveal UM)	[19, 34, 35, 94, 95, 96, 97, 98, 99, 100, 101, 102, 103, 111, 118, 119]

## 3 Laser and Photo‐Induced Therapies for UM

Laser‐ and light‐based therapies are innovative treatment approaches in the management of UM, particularly for small tumors or cases where other treatment modalities (radiation or surgery) may not be clinically feasible. They encompass a range of methods that rely on the interaction between light energy and tumor tissues, aiming at tumor reduction or destruction.

### 3.1 Major Types of Treatments and Their Mechanisms

Photocoagulation was one of the first methods to use a laser source for the treatment of UM, initially implemented with diode lasers and various types of arc lasers, such as xenon, argon (488 and 514 nm), and krypton lasers (647 nm) [81, 82]. Photocoagulation is primarily indicated for small tumors (basal diameter: 4.5–10 mm; thickness: 1.5–3.5 mm) with a well‐defined location, where the use of a focused laser beam offers both technical and clinical advantages [83, 84]. The fundamental mechanism underlying photocoagulation is photothermal in nature. Laser‐emitted light energy is absorbed by tissue chromophores within the tumor, predominantly melanin, and by hemoglobin contained in the intratumoral vasculature. Upon absorption, the light energy is converted into thermal energy (heat), resulting in localized heating of the tumor. This increase in temperature can trigger two primary mechanisms: tissue coagulation, derived from protein denaturation and aggregation, and thermal necrosis of tumor cells (thermodestruction). These combined effects result in structural and functional alteration of the melanoma; however, the overall efficacy of photocoagulation is constrained by the laser beam's penetration depth and the dimensions of the treated lesions [19, 85].

In response to the limitations of laser photocoagulation, particularly regarding penetration depth and tumor control, TTT emerged and quickly replaced photocoagulation as the main laser‐based modality for the treatment of UM. It was developed and implemented primarily during the 1990s–2000s by Oosterhuis and Shields [86, 87, 88]. TTT is based on the use of a NIR laser (most commonly a standardized 810 nm diode laser), which allows deeper tissue penetration than the visible lasers used in photocoagulation, applied through a dilated pupil onto the surface of the choroidal tumor [88, 89, 90]. Its principle is based on controlled photothermia or hyperthermia therapy, aiming to gradually raise the tumor's temperature to 45–60°C to induce thermal necrosis of the tumor cells, while minimizing damage to the surrounding healthy tissues [88, 89, 90, 91]. It has become the most widely adopted laser‐based therapeutic modality for UM malignancy. Furthermore, TTT was long employed as an adjuvant to radiotherapy; however, this combination is now rarely applied [32, 92, 93].

PDT provides a targeted, nonthermal light‐mediated modality, with high spatial selectivity, intended to spare healthy tissues and preserve patients’ visual function [34, 94, 95]. It involves the use of light in combination with a systemically administered photosensitizer that preferentially accumulates in tumor cells or within the tumor vasculature. The tumor region is irradiated with a defined red‐light wavelength (630–690 nm), selected to minimize melanin absorption, resulting in electronic excitation of the photosensitizer. The excited photosensitizer subsequently transfers its energy to the surrounding oxygen, generating cytotoxic reactive oxygen species (ROS), which induce direct tumor cell death via apoptosis or necrosis. It simultaneously causes vascular occlusion within the tumor, leading to localized ischemia and ultimately cell death [96, 97, 98]. Several photosensitizers are used in PDT for UM, the most widely employed being hematoporphyrin (HpD), CASPc (a second‐generation porphyrin), and verteporfin (VP), also known as benzoporphyrin derivative monoacid ring A (BPD‐MA; commercial name Visudyne) [34, 35, 99, 100]. PDT is generally performed according to the TAP protocol (Treatment of Age‐related Macular Degeneration with Photodynamic Therapy) [94, 101] or one of its variants [102]. The TAP protocol involves intravenous administration of VP at a dose of 6 mg/m^2^ (body surface area) over 10 min. Five minutes after infusion, the target tissue is irradiated with red laser light (λ = 690 nm) at an intensity of 600 mW/cm^2^ for 83 s, corresponding to a fluence of 50 J/cm^2^. The number of treatment sessions varies from 1 to 3 depending on the patient's clinical response [19, 35, 101, 103]. Better tissue penetration and lower phototoxicity are achieved with VP compared to other photosensitizers. Finally, the fluence and number of spots are adjusted according to the tumor's thickness, pigmentation, and location, as well as patient‐specific characteristics [19, 34].

### 3.2 Limitations of the Different Therapeutic Modalities

The laser‐based treatments described in Section 3.1, namely, photocoagulation, TTT, and PDT, are employed in the management of primary UM. Nevertheless, they are associated with significant limitations including tumor control efficacy, the risk of recurrence, and potential adverse effects.

Photocoagulation, historically one of the earliest treatments for UM, is now considered only in specific cases. One of the main limitations lies in the difficulty of confirming complete tumor eradication. Despite apparent initial efficacy on conventional imaging examinations (B‐scan ultrasonography, fluorescein angiography, etc.), numerous recurrences have been reported [83, 104]. Tumor cells may develop resistance and invade the sclera (the outer white layer of the eyeball), promoting recurrence [105, 106]. Furthermore, photocoagulation is technically limited to small tumors [15]. Limited laser penetration prevents effective destruction of thicker lesions, also increasing recurrence risk [19, 84]. Tumor location also affects efficacy, lesions must be sufficiently distant from the fovea to avoid vision loss [15]. Finally, melanoma pigmentation strongly affects laser energy absorption, limiting tissue destruction in depth, and further compromising the possibility of complete tumor elimination [84].

TTT, although commonly used to treat UM, remains associated with significant limitations, notably the restricted thermal penetration due to tumor thickness. Indeed, TTT is effective only for thin tumors (<4.5 mm). Deeper tumor cells are not accessible to infrared energy, preventing complete tumor destruction and increasing the risk of residual disease. Clinically, this limitation is reflected in a notable recurrence rate. Early recurrences occur in approximately 9% of cases, rising to nearly 23% over long‐term follow‐up (from one year onward), indicating insufficient tumor control [107, 108]. Tumor location also influences the efficacy of TTT. Lesions located near the optic disc or close to the fovea are particularly difficult to treat, because heat delivery is constrained by the proximity to these sensitive structures, thereby also increasing the risk of recurrence and severe visual impairment [88, 107]. In addition, peripheral tumors present technical challenges, such as precise targeting [31]. In some cases, secondary cellular proliferation induced by thermal stress may also occur [31, 90, 109]. Thus, despite its limitations, TTT remains a relevant therapeutic option for small tumors selected according to strict criteria, as its efficacy strongly depends on tumor and anatomical features [110].

PDT also presents several major limitations that reduce its clinical effectiveness. One of the most critical limiting factors is melanin pigmentation. Melanin strongly absorbs visible light, including the red wavelengths (630–690 nm) used to activate many photosensitizers in PDT [111]. The melanin‐mediated absorption decreases the amount of light reaching the photosensitizer and consequently reduces the production of ROS, thereby rendering highly pigmented tumors less responsive to PDT compared with weakly pigmented or amelanotic ones [34, 112, 113, 114]. The effectiveness of PDT also depends on the choice of the photosensitizer. Phthalocyanines, which are activated in the NIR range (700–800 nm) [115], maintain high therapeutic efficacy in pigmented tissues, in contrast to second‐generation porphyrins such as VP, activated at 690 nm and therefore markedly affected by melanin absorption. Several studies have reported the absence of complete tumor destruction in highly pigmented melanomas, even when high light fluences are applied [96]. Furthermore, PDT is also generally restricted to small (thickness ≤ 3 mm), well‐localized tumors, as the limited optical penetration prevents homogeneous treatment of large or diffuse lesions.

Beyond these limiting factors, the efficacy of PDT is highly dependent on the individual features of the tumors, including thickness, basal diameter, localization, and vascularization, leading to highly variable responses from one patient to another. Clinical studies consequently report recurrence rates ranging from 16% to more than 60% in long‐term follow‐up (with a mean of 60 months), as well as approximately 10% of melanoma cases in which no therapeutic response to PDT was observed. These findings highlight the challenge of achieving durable tumor control with PDT [95, 97, 116]. Collectively, these adverse outcomes explain the relatively limited use of PDT compared with other therapeutic modalities for UM, despite its important therapeutic potential.

## 4 Phototherapies Combined with Nanoparticles

### 4.1 Interest of Nanoparticles in Ophthalmology

#### 4.1.1 Ocular Barriers and Ophthalmic Delivery Challenges

The eye is a highly specialized organ with a complex anatomy, several ocular barriers (blood–aqueous barrier (BAB), blood–retinal barrier (BRB), conjunctiva, cornea, efflux pumps, etc.), and heightened sensitivity [120, 121, 122]. Ophthalmic treatments are often limited by rapid elimination of active ingredients (via the tear film, systemic absorption, conjunctival uptake, etc.) and by poor penetration into deeper ocular structures such as the choroid and ciliary body, due to the low permeability of the retinal pigment epithelium [120, 121]. Moreover, ophthalmic treatments often lack precision due to suboptimal routes of administration, such as topical or systemic delivery routes (with less than 5% of the administered dose reaching the targeted intraocular tissues via the topical route). This limits the availability of active ingredients to the site of action and may induce toxicity in healthy tissues [123, 124, 125]. Within this context, defined by the presence of ocular barriers and the inherent limitations of conventional ophthalmic therapeutic treatments, the use of nanoparticles (NPs) is particularly justified and represents a promising alternative for treating ocular diseases.

The International Union of Pure and Applied Chemistry defines NPs and nanomaterials as materials with at least one dimension in the nanometer range (1–100 nm) [126]. NPs allow for either active tumor targeting, via a specific targeting agent, or passive targeting, by means of the enhanced permeability and retention (EPR) effect, thus reducing systemic exposure [127, 128, 129]. They can carry and locally activate therapies via stimuli (pH, temperature, light) [127, 130, 131, 132, 133], are less immunogenic when non‐virus‐derived, and support advanced treatments such as nucleic acid and immunotherapy delivery [134, 135, 136, 137, 138, 139, 140]. A variety of NPs are proposed in the fields of oncology/nanomedicine, from three main families: [127, 141, 142] (i) organic NPs, most commonly composed of lipids (e.g., liposomes, lipid NPs (LNPs)) [143, 144], extracellular vesicles [145] or polymers (e.g., nanomicelles, polymeric NPs) [146, 147, 148], (ii) inorganic NPs, made of metals, oxides, or semiconductors [149, 150, 151, 152] and (iii) hybrid NPs typically combining an inorganic core with an organic shell, or vice versa [153, 154].

#### 4.1.2 Specific Advantages of NPs in Ophthalmology

NPs can traverse ocular barriers, facilitate the delivery of a wide range of molecules and biomolecules (including therapeutic agents, photosensitizers, proteins, nucleic acids, etc.), and improve the stability and biocompatibility of active compounds [155, 156].

Furthermore, NPs can adhere to the ocular surface (mostly polymeric NPs presenting mucoadhesive properties), enhancing their biodistribution. Nonetheless, the efficiency of therapeutic delivery, as well as the resulting toxicity and overall biodistribution, is influenced by multiple NP properties, including composition, size, shape, surface charge, hydrophilicity or hydrophobicity, biodegradability, and by the route of administration employed [122, 157, 158, 159, 160]. Examples of the impact of NP properties on their distribution, for different administration routes, are summarized in Table 3.

**TABLE 3:** Administration route and NPs size impact on distribution in ophthalmology.

Administration route	NPs size	Behavior and distribution	Typical clinical applications	Examples of nanosystems	Challenges	Refs.
**Topical** **(drops, gels)**	<10 nm	Drug penetration limited by the tear film and the corneal barrier	Glaucoma medication, antibiotics, anti‐inflammatories	Dendrimers (PAMAM)	Cellular toxicity, low retention	[161, 162, 163, 164]
	10–50 nm	Better corneal penetration	Treatments of infections (keratitis) or inflammation (anterior uveitis)	Nanomicelles	Clearence by tear film, blink of eyes, corneal barrier, efflux pumps	[122, 125]
	50–200 nm	Good retention on cornea and conjunctiva, transepithelial penetration	Delivery of anti‐inflammatory medications, antioxidants and anti‐VEGF antibody (vascular endothelial growth factor)	Nanoemulsions (Restasis), liposomes (Lacrisek), polymeric NPs (poly(lactic‐*co*‐glycolic acid) (PLGA), polycaprolactione (PCL))	Dilution by tear renewal, need for sustained‐release systems, conjunctival drainage	[122, 125, 160, 164, 165, 166]
	200–500 nm	Difficulty crossing cornea (limited penetration < 150 nm), good surface adhesion	Controlled release of antibiotic drugs for infections, glaucoma, uveitis, cataract	Nanosuspension (e.g., hydrocortisone)	Potential aggregation, low penetration, conjunctival drainage	[122, 125, 167, 168, 169, 170]
	>500 nm	Blocked by tear film and corneal barrier, good for long retention on ocular surface	Prolonged release and improved retention on the ocular surface, glaucoma	Polymeric microparticles	Clearance	[171, 172]
**Intravitreal**	<50–100 nm	Rapid diffusion in vitreous, risk of retinal leakage	Delivery of anti‐VEGF antibody (Aflibercept) or gene therapies	Cysteine‐Lysine_30_‐Poly(ethylene glycol) CK/PEG‐DNA polyplex, polymeric nanocarriers	Retinal toxicity, rapid clearance	[122, 160, 173]
	100–200 nm	Good vitreous distribution, retinal penetration (if neutral / negative charge)	Retinal detachment, excitotoxicity, optic nerve crush	Polymeric NPs (chitosan, PGLA, PEG) nanoemulsions, niosomes, lipids NPs	Risk of aggregation in vitreous (depending of charge)	[122, 160, 174, 175]
	200–500 nm	Slow diffusion in vitreous, prolonged retention	Delivery of neuroprotective agents, anti‐VEGF antibody , glaucoma, diabetic retinopathy	Polymeric NPs, hydrogels, microparticles,	Aggregation, mechanical obstruction risk	[122, 160, 174, 175]
	>500 nm	Immobilization in vitreous, poor retinal penetration	Not suitable (except for local deposits)	Polymeric microparticles (e.g., PGLA microspheres, implants	Invasiveness, aggregation, risk of retinal detachment	[176, 177, 178]
**Subconjunctival**	10–100 nm	Rapid penetration into sclera, good option to target choroid and external retina	Prevention of corneal graft rejection (dexamethasone), anti‐VEGF.	Polymeric NPs (e.g., pRNA)	Conjunctival lymphatic drainage Absorption via conjunctival blood capillaries	[179]
	100–500 nm	Increased permeability for NPs larger than 200 nm, local retention, less scleral penetration, possible prolonged release	Corneal graft rejection, autoimmune uveitis, brucellosis	PLGA‐PEG NPs, hydrogels, antigen complex	Rapid elimination, permeability	[180, 181]
	>500 nm	Prolonged local retention at the injection site, formation of a subconjunctival particulate deposit	Slow‐release drug depots, uveitis	Polymerics microparticles	Risk of local inflammation, fibrosis, low degradation (deposit)	[182, 183]
**Suprachoroidal**	20–100 nm	Rapid lateral diffusion into the choroid, retina, and retinal pigment epithelium	Delivery of β‐blockers or carbonic anhydrase inhibitors (glaucoma), delivery of anti‐VEGF antibody , antioxidants	Polymeric NPs (polystyrene), viscous solutions (hyaluronic acid).	Complex technique (high vascularization, possible acute retinal lesion), bleeding risk	[184]
	100–500 nm	Dense but limited distribution, prolonged retention in the suprachoroidal space		Polymeric NPs and microparticles (e.g., polystyrene)	Requires precise injection	[185]
	>500 nm	Local immobilization and poor diffusion	AMD, macular edema, delivery of anti‐VEGF antibody	Microparticles (e.g., microneedles) Solid implant	Fibrosis risk	[185]
**Systemic**	<50 nm	Potential to cross blood‐retinal barrier, Significant diffusion is possible, requiring targeting to overcome the barrier, EPR effect	Systemic treatments with retinal targeting (e.g., autoimmune uveitis)	Polymeric NPs (PEGylated), liposomes	Low efficacy without ligands, systemic toxicity	[122, 160, 186]
	50–200 nm	Prolonged circulation, retinal penetration via EPR effect, accessibility to choroid		Polymeric NPs (e.g. poly(lactic acid)–poly(ethylene glycol) (PLA‐PEG)), dendrimers	Hepatic/splenic clearance	[122, 124, 160]
	>300 nm	Rapid elimination by reticuloendothelial system	Not suitable	Microparticles	Low ocular bioavailability, hepatic/splenic clearance	[122, 124, 160]

Several nanomedicine‐based therapeutics have already received FDA approval for the treatment of a range of ocular diseases, including glaucoma, uveitis, ocular inflammation or infection, and age‐related macular degeneration (AMD). Notable examples include Visudyne, a liposomal formulation of VP indicated for PDT in AMD, approved by the FDA (2000) [187]; Triesence [188], a microneedle‐based delivery system for triamcinolone acetonide, approved for multiple indications such as uveitis, sympathetic ophthalmia, and temporal arteritis (2007), and Xelpros, an oil‐in‐water microemulsion formulation approved for the treatment of glaucoma [189].

Numerous nano‐based drug delivery systems are currently under clinical evaluation. For instance, OCS‐01 is a topical eye drop‐type NP formulation designed for the delivery of dexamethasone in the treatment of diabetic macular edema (DME) and is presently in Phase III clinical trials (recruiting) [190]. Another example is KSI‐301, an anti‐VEGF (vascular endothelial growth factor) antibody conjugated to a biopolymer to enable sustained intraocular drug delivery, which has been investigated in Phase III clinical studies for retinal vein occlusion, DME, and AMD [191]. In parallel, an increased number of nanosystems is being actively explored at the preclinical stage, including silica NPs [192, 193], gold NPs, [194, 195] quantum dots [196], and iron oxide NPs [197], among many others.

### 4.2 Interest of Phototherapies Combined With NPs for UM

As previously highlighted in Section 3 on laser‐based therapies, conventional treatment modalities for UM present significant limitations, many of which may be addressed by phototherapeutic strategies, particularly when combined with nanotechnology‐based systems.

Phototherapy, primarily including PDT and photothermal therapies (PTT), offers a localized, rapid, tunable, and noninvasive effective approach amenable to repeated application when needed, particularly suitable for ocular diseases, with direct optical access to the eye tissues [198, 199, 200]. However, it is associated with limited penetration of UV and visible light, lack of tumor selectivity, reduced efficacy in hypoxic TME, especially for PDT, insufficient accumulation of photosensitizers, and potential irradiation‐related side effects such as burns, inflammation, or tissue damage [198, 199, 201]. In light of this context and the associated challenges, nanomedicine through the rational design of therapeutic nanosystems has emerged as a pivotal strategy in advancing phototherapy, and may offer distinct advantages to enhance the efficacy and specificity of the treatments intended for UM [201].

In this section, we highlight how engineered NPs can function as multifunctional theranostic platforms capable of simultaneously enhancing photosensitizer bioavailability, improving light conversion efficiency, remodeling the TME, stimulating antitumor immunity, and reducing off‐target ocular toxicity. In addition, it is worth mentioning here that most NP‐based therapeutic approaches for UM focus on overcoming barriers associated with posterior‐segment delivery, including the blood–retinal barrier, intratumoral penetration, local retention, and targeted accumulation within the TME. Current phototherapeutic strategies are generally based on systemic, intravitreal, periocular, or suprachoroidal administration rather than topical delivery. Mucoadhesive NPs may represent a promising future strategy for noninvasive ocular drug delivery; however, their direct applicability to NP‐based phototherapy for UM remains less established compared with other delivery approaches specifically designed for posterior intraocular targeting. Mechanistic roles of NP systems in overcoming therapeutic challenges associated with UM are summarized in Table 4.

**TABLE 4:** NP‐mediated strategies potentially useful for addressing key limitations of UM therapy.

Therapeutic limitations in UM	NP‐enabled strategies	Mechanistic basis	Refs.
**Insufficient intratumoral photosensitizer accumulation**	Encapsulation within liposomes, polymeric NPs, mesoporous silica NPs, dendrimers, and lipid‐based nanocarriers	Free photosensitizers exhibit poor aqueous solubility, rapid systemic elimination, aggregation‐induced quenching, and limited tumor selectivity. NP encapsulation increases loading capacity and colloidal stability while preventing premature degradation and self‐aggregation. Surface functionalization with targeting ligands such as RGD peptides, folate receptors, transferrin, or monoclonal antibodies facilitates receptor‐mediated endocytosis and selective accumulation within UM cells, thereby enhancing local photosensitizer concentration and improving photodynamic quantum efficiency.	[202, 203, 204, 205, 206, 207, 208, 209, 210, 211]
**Restricted tissue penetration and suboptimal activation wavelength**	NIR ‐responsive nanostructures including gold nanostructures, copper sulfide NPs, carbon nanomaterials, and upconversion NPs	Conventional photosensitizers predominantly absorb visible light within the range of approximately 630–690 nm, limiting photon penetration through melanin‐rich ocular tissues. NPs capable of NIR absorption or photon upconversion shift excitation into the biological optical window (approximately 700–1100 nm). Upconversion NPs can additionally convert low‐energy NIR photons into shorter wavelengths capable of activating conventional photosensitizers, thereby increasing therapeutic depth and improving energy transfer efficiency within intraocular lesions.	[211, 212, 213, 214, 215]
**Tumor hypoxia limiting ROS production**	Oxygen‐generating and oxygen‐carrying NPs including MnO_2_ NPs, catalase‐loaded systems, and perfluorocarbon nanocarriers	The efficacy of PDT is critically dependent on oxygen availability as singlet oxygen generation requires molecular O_2_. Hypoxic regions within UM reduce ROS production and induce treatment resistance. Catalytic NPs locally decompose endogenous hydrogen peroxide into oxygen, while perfluorocarbon‐based systems act as oxygen reservoirs. Enhanced oxygen availability improves photochemical reaction kinetics and amplifies intracellular oxidative stress, resulting in greater tumor cell apoptosis and necrosis.	[208, 210, 211, 216, 217, 218, 219]
**Weak immune activation resulting from ocular immune privilege**	Immunomodulatory NPs incorporating adjuvants, cytokines, immune checkpoint inhibitors, or nucleic acid therapeutics	The ocular microenvironment exhibits immune‐suppressive characteristics that attenuate antigen presentation and cytotoxic T‐cell activation. NP‐mediated induction of immunogenic cell death promotes release of danger‐associated molecular patterns (DAMPs), including calreticulin, ATP, and HMGB1, which facilitate dendritic cell maturation and antigen cross‐presentation. Simultaneous delivery of immunostimulatory molecules may reverse local immune suppression and promote systemic anti‐tumor responses, potentially generating an abscopal effect against metastatic disease.	[206, 207, 220, 221, 222, 223, 224, 225]
**Limited drug penetration through BOB**	Intravitreal, periocular, or suprachoroidal NP administration with sustained‐release formulations	BRB and BAB substantially limit intraocular penetration of systemically administered agents. Local administration of NP formulations bypasses these anatomical restrictions and provides prolonged therapeutic retention through controlled release kinetics. Increased intratumoral bioavailability can be achieved while minimizing systemic exposure and reducing dose‐dependent adverse effects.	[161, 208, 226, 227, 228]
**Retinal toxicity and collateral tissue injury**	Stimuli‐responsive and tumor‐selective NP systems	Nonspecific laser irradiation and photothermal treatment can induce damage to surrounding retinal and optic tissues due to uncontrolled energy deposition. Stimuli‐responsive NPs designed to respond to local pH alterations, elevated ROS concentrations, enzyme activity, photoresponsive or external triggers can selectively release therapeutic payloads within the TME. Spatially controlled activation reduces collateral damage and can preserve normal retinal architecture and visual function.	[206, 207, 208, 209, 212, 225, 229, 230, 231, 232]

From a mechanistic perspective, NP‐mediated treatment fundamentally shifts therapeutic design from a predominantly passive energy‐delivery paradigm toward a precision‐guided theranostic framework. In conventional phototherapeutic approaches, efficacy often depends on increasing laser power density to compensate for insufficient tissue penetration and heterogeneous photosensitizer distribution. However, this strategy is problematic in UM because the confined intraocular space and variable melanin content increase the risk of thermal injury and retinal damage. Multifunctional NPs instead amplify therapeutic responses at the tumor site by increasing local drug concentration, improving optical conversion efficiency, modulating oxygen availability, and initiating immune activation while simultaneously reducing the external energy requirements. Consequently, NP systems may substantially improve therapeutic selectivity, reduce ocular toxicity, and overcome several biological and physicochemical barriers that currently limit clinical outcomes in UM management.

### 4.3 Main Combined NP‐Phototherapy Strategies Relevant to UM

UM represents a particularly suitable target for phototherapy, owing to its accessibility to light irradiation, making photoactivation‐based strategies especially promising. Despite significant progress and examples of nanosystems developed in preclinical studies, none have yet been approved by the FDA nor integrated into clinical practice for the treatment of UM.

To date, only one approach has entered clinical trials. This system, AU‐011, is based on virus‐like particles (VLPs) derived from human papillomavirus capsid proteins (L1/L2) which self‐assemble to form a multivalent structure. The VLPs are conjugated to the NIR photosensitizer IRDye 700DX (IR700) phthalocyanine, activated upon 690 nm NIR irradiation. The system selectively targets tumor cells by binding to surface proteoglycans (heparan sulfate proteoglycans (HSPGs)), absent in healthy tissues. Preclinical studies evaluated intravitreal and intravenous administration [233], whereas clinical trials employ the suprachoroidal route [234]. AU‐011 acts by binding to tumor cells via HSPGs, followed by light activation that induces ROS generation and localized heating, leading to cell membrane rupture and cell death. Its light‐dependent activation and requirement for direct binding limit off‐target effects, reducing systemic toxicity. The multivalent VLPs structure also enables the delivery of a wide range of cytotoxic molecules, enhancing treatment efficacy. This approach offers precise local tumor control while preserving surrounding ocular structures, making AU‐011 particularly attractive for the treatment of primary UM. It is especially suited for early‐stage lesions or tumors, and may also stimulate antitumor immune response. First described by Kines et al. [233] as a light‐activated targeted therapy, AU‐011 has since shown the ability to induce immunogenic cell death (ICD) (Ma et al., LUMC, DRUM) [235]. Its clinical development, led by Aura Biosciences, has completed Phases Ib/II trials and is currently in Phase III, highlighting its strong potential for treating UM and possibly other tumors [234].

Preclinical research in UM has investigated a limited number of NP‐based systems, with nine reported to integrate PDT and/or PTT, alone or in combination. These nanoplatforms are also frequently combined with immunotherapy and/or chemotherapy, reflecting the emerging yet still relatively unexplored potential of nanophototherapeutic approaches in UM.

The first study was reported in 2021 by Kim et al. [224], who developed an effective treatment for UM, which is typically resistant to immunotherapy and prone to metastasis. The researchers proposed an immunotherapeutic approach, in combination with local PDT, using a pluronic F‐127 nanophotosensitizer embedding Ce6 and iodine‐rich diatrizoic acid (FIC‐PDT), Rho‐associated kinase (ROCK) inhibition via the clinically approved drug ripasudil, and immune checkpoint blockade (ICB) with anti‐PD‐L1 (Programmed Death‐Ligand 1) antibodies. FIC‐PDT triggers ICD in melanoma cells, evidenced by release of damage‐associated molecular patterns (DAMPs) such as calreticulin (CRT), High Mobility Group Box 1 (HMGB1), Heat Shock Protein 70 (HSP70), and adenosine triphosphate (ATP). ICD leads to recruitment and activation of antigen‐presenting cells (APCs), such as dendritic cells (DCs) and macrophages. Ripasudil improves the ability of APCs to engulf dying tumor cells by inhibiting ROCK signaling. This boosts antigen presentation and primes cytotoxic CD8^+^T cells. Combined FIC‐PDT and ripasudil treatment transforms the “cold” TME (immunosuppressive) into a “hot” TME (immunoreactive). This increases infiltration of CD8^+^ cytotoxic T cells and upregulates PD‐L1 expression, making tumors more responsive to ICB therapy. Here, the triple therapy (FIC‐PDT + ripasudil + anti‐PD‐L1) not only reduced primary tumor burden but also suppressed distant (secondary) tumors in a bilateral B16F10 mouse melanoma model. The treatment led to increased CD103^+^ DCs and CD8^+^ T cells in both primary and metastatic sites. Even at low doses, triple therapy significantly reduced intraocular tumors and prevented lung metastases in an orthotopic model of B16F10 melanoma. Immunohistochemistry confirmed increased CD8^+^ T cell infiltration and proliferation, as well as favorable T cell/Treg ratios. The study introduces a clinically translatable strategy that induces in situ vaccination against UM through immunogenic clearance, sensitizes tumors to ICB, achieves systemic antitumor immunity capable of inhibiting metastasis, the major challenge in UM therapy. This approach holds potential for addressing the current unmet clinical need in treating UM and possibly other “cold” tumors resistant to conventional immunotherapy.

**FIGURE 2:**
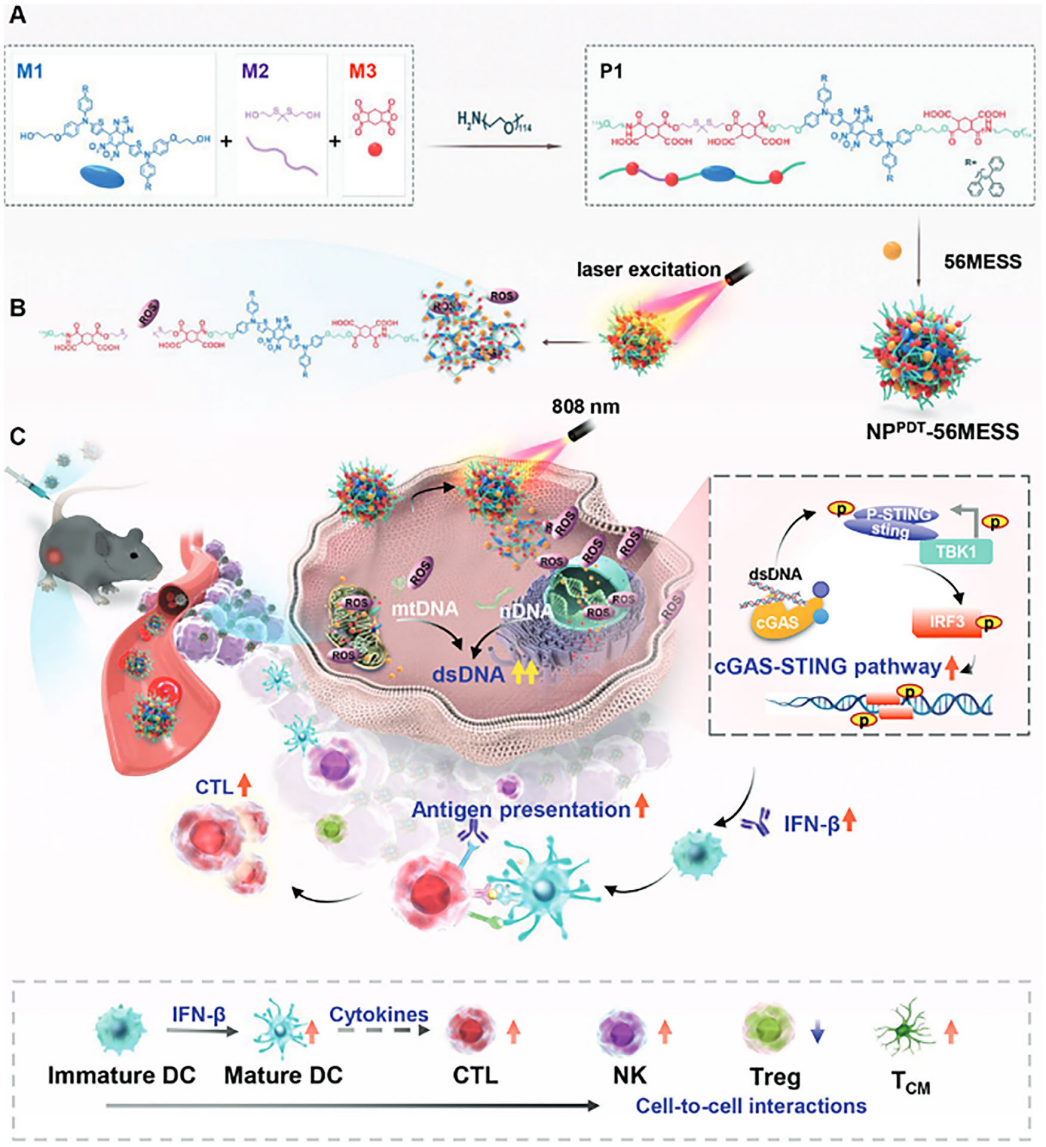
Schematic illustration of cGAS‐STING pathway activation and anti‐tumor immunity induction via NPPDT‐56MESS + L. A) Preparation of NP_PDT_‐56MESS via self‐assembly of P1 with 56MESS, M1: AIE monomer 7 based on a TPE‐triphenylamine‐thiophene‐dinitrobenzobisthiadiazole scaffold bearing two terminal hydroxyethyl groups/M2: 2,2′‐(Propane‐2,2‐diylbis(sulfanediyl))bis(ethan‐1‐ol)/M3: 1,2,4,5‐cyclohexanetetracarboxylic dianhydride and P1: PEGylated polyurethane containing a TPE‐triphenylamine‐thiophene‐dinitrobenzobisthiadiazole AIE photosensitizer unit. B) NP_PDT_‐56MESS produces ROS and releases 56MESS upon excitation by a NIR 808 nm laser. C) NPPDT‐56MESS + L increases dsDNA in the cytoplasm, which activates the cGAS‐STING pathway. Subsequently, IFN‐β is released by tumor cells to promote DC maturation, inducing an anti‐tumor immune response in vivo. Reproduced with permission [227]. Copyright 2023, Wiley.

Following these findings, other studies leveraged the PDT‐immunotherapy combined approach to prevent metastasis and tumor recurrence. Tao et al. [225] designed an amphiphilic polymer that self‐assembles into NPs. This polymer is composed of three monomers: a photosensitizer, a ROS‐sensitive thioketal linker, and a carboxylic acid‐containing linker capable of complexing 56MESS. The polymer was loaded with 56MESS to form NP^PDT^‐56MESS NPs. Under laser irradiation at 808 nm, the particles generate ROS, decompose, and release 56MESS. After endocytosis into OCM‐1 cells, ROS and 56MESS synergistically damage DNA and mitochondria, thereby increasing the amount of cytoplasmic double‐stranded DNA. This activates the cGAS‐STING pathway. The cGAS‐STING pathway is an innate immune signaling pathway that triggers antitumor immunity, but existing STING agonists lack tumor‐targeting capabilities. 56MESS is a platinum‐based DNA intercalator that causes significant DNA damage (Figure 2). Activation of the cGAS‐STING signaling pathway increased IFN‐β and IL‐6 secretion in the supernatant of OCM‐1 cells. Co‐incubation of treated B16F10 cells with bone marrow‐derived DC induced DC maturation and immune activation. Metabolomic analyses revealed altered energy metabolism and disrupted nucleotide synthesis. In vivo safety and distribution studies, performed by caudal intravenous injection in OCM‐1 tumor‐bearing mice, showed that the NPs accumulated specifically at the tumor site. Lower systemic toxicity than that of free 56MESS (which causes hepatotoxicity) was observed in healthy KM mice. The in vivo therapeutic effects were studied in murine models of UM. NP^PDT^‐56MESS and laser irradiation inhibited tumor growth and induced DNA damage, thus demonstrating the efficacy of the NPs. The efficacy of NPPDT‐56MESS was then evaluated in mice bearing B16F10 tumors. Irradiation of the NPs in the tumors induced efficient activation of the cGAS‐STING pathway in vivo. The immune response was then analyzed. DCs maturation was observed. In addition, infiltration of NK cells and CD8^+^ T lymphocytes, associated with a reduction in regulatory T cells (Tregs) in the tumors, was demonstrated. Long‐term immunity was studied. The combination of NP^PDT^‐56MESS and laser‐induced immunological memory through the expansion of central memory T cells. Tumor recurrence and metastasis were prevented. A survival rate of 80% was achieved at day 60 in mice reexposed to the treatment.

**FIGURE 3:**
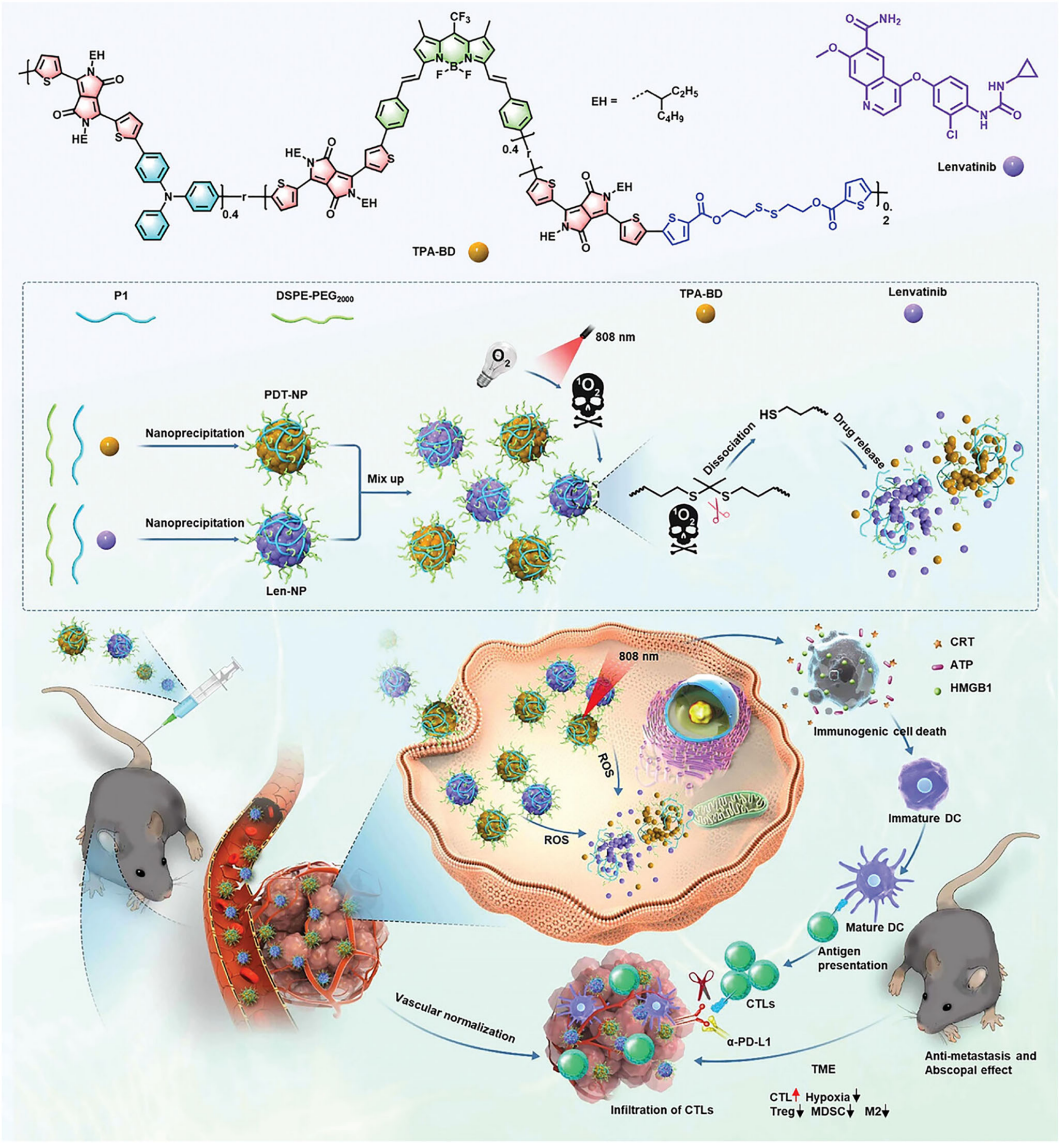
Schematic illustration of NIR‐II theranostic Combo‐NP for photodynamic‐immunotherapy via normalizing blood vessels. A NIR II fluorescent biodegradable pseudo conjugate polymer with disulfide bonds was designed (TPA‐BD). TPA‐BD was then adopted to deliver a multi‐targeted tyrosine kinase inhibitor, ie., Lenvatinib to normalize the tumor vessels, aiming to alleviate tumor hypoxia and improve the PDT efficiency. Subsequently, the normalized vasculature facilitated the infiltration and migration of CTLs into the TME while decreased the recruitment of immunosuppressive cells (MDSC, Treg, M2). Furthermore, combination of ‐α‐PD‐L1 mAb with Combo‐NP induced systemic antitumor‐mediated abscopal effect and inhibited cancer metastasis on mice cancer models. Reproduced with permission [208]. Copyright 2023, Wiley.

Furthermore, these studies raised an additional challenge, PDT is effective and minimally invasive, but its efficacy is limited by tumor hypoxia induced by abnormal vascularization. Hypoxia reduces the production of ROS and limits immune cell infiltration, thus compromising PDT and immunotherapy effects. Lenvatinib is a multikinase inhibitor that can remodel abnormal tumor vessels, mitigate hypoxia, and enhance immune responses [236, 237]. In an interesting study, the group of Zheng et al. [206] developed a novel NP system (Combo‐NP) incorporating two types of NPs, one of which encapsulates Lenvatinib. A first polymer, triphenylamine–benzodithiophene conjugated polymer (TPA‐BD) (fluorescent in the NIR II, biodegradable due to its disulfide bridges, and generating ROS under laser irradiation at 808 nm), was designed for PDT and co‐assembled with 1,2‐distearoyl‐sn‐glycero‐3‐phosphoethanolamine‐poly(ethylene glycol) 2000 (DSPE‐PEG2000) to form PDT NPs (PDT‐NP). A second ROS‐sensitive polymer encapsulating Lenvatinib is a drug intended for vascular normalization. This system was then treated with DSPE‐PEG2000 to obtain Len‐NP. Combined NPs (Combo‐NP) were obtained by mixing Len‐NP and PDT‐NP in specific proportions. The anticancer mechanism is as follows: light irradiation induces the generation of ROS by TPA‐BD, leading to the direct destruction of tumor cells and the induction of immunogenic cell death. ICD triggers the release of DAMPs (ATP, HMGB1, CRT), the maturation of DCs, and the activation of cytotoxic T lymphocytes (CTLs). ROS also trigger the release of Lenvatinib, which normalizes vascularization, reduces hypoxia, and promotes CTL infiltration. Combo‐NP and a PD‐L1 ICI enhanced systemic immunity and prevented metastasis. These NPs were efficiently internalized into UM (OCM‐1) and B16F10 melanoma cells and generated significantly more ROS than controls after laser irradiation. NIR‐II fluorescence was used to monitor internalization. Laser irradiation induced apoptosis in OCM‐1 cells (36.2%) compared to the control (phosphate‐buffered saline (PBS), 3.1%), and similar results were obtained in B16F10 cells. In 3D spheroids of B16F10 cells, laser irradiation induced higher rates of apoptosis and cell death than PDT‐NP or Len‐NP. Irradiation promoted the induction of ICD with increased exposure to CRT, ATP release, and HMGB1 efflux in both B16F10 and OCM‐1 cells. Laser irradiation also promoted DCs maturation (37% vs. 18% for PBS). In vivo studies were conducted. Len‐NP, PDT‐NP, and Combo‐NP were intravenously injected into KM mice. No significant toxicity was observed. Body weight remained stable, blood biochemistry was normal, and no organ damage was noted. An OCM‐1 subcutaneous tumor model was created in mice. Cy7.5‐labeled Combo‐NP accumulated efficiently at the tumor site (fluorescence peak at 24 h, still visible at 48 h). In OCM‐1 xenografts, the combination of Combo‐NP and laser reduced tumor weight by a factor of 12 compared to PBS. In B16F10 models, the combination of Combo‐NP and laser induced almost complete tumor reduction, far surpassing controls. Histological studies revealed marked necrosis and apoptosis in tumor tissues. An increase in pericyte coverage (91.2%) and a reduction in hypoxia (HIF‐1α expression analyzed) were observed. Normalization of vascularization improved cytotoxic CTL infiltration and reduced the number of immunosuppressive cells (Tregs, myeloid‐derived suppressor cells (MDSCs), M2 macrophages). Combo‐NP and a laser increased CD8+ T lymphocyte infiltration into tumors (38.9% vs. 28.8% for PDT with Combo‐NP and laser), increased the number of mature DCs in tumors and lymph nodes, and significantly reduced immunosuppressive cell populations (e.g., regulatory T lymphocytes (Tregs) decreased from 54% to 18.8%) (Figure 3). PDT and Combo‐NP induced time‐dependent overexpression of PD‐L1 in B16F10 and OCM‐1 cells, as well as in tumor tissues, thus preparing tumors for ICB. In bilateral tumor models, Combo‐NP and a laser, along with an anti‐PD‐L1 agent, virtually eliminated both primary and metastatic tumors. Powerful abscopal effects were observed, inhibiting lung metastases (no visible lesions were observed after treatment). Immune profiling revealed greater cytotoxic T‐cell infiltration and a lower number of immunosuppressive cells than with Combo‐NP alone.

Another notable example illustrating the PDT‐immunotherapy combined effect in UM treatment is a hyaluronic acid‐based nanosystem targeting CD44 (Cluster of Differentiation 44) and encapsulating verteporfin (HANP/VP). VP is an FDA‐approved photosensitizer for eye diseases and tested in several cancers. VP possesses a dual function, it generates ROS under PDT and inhibits YAP‐TEAD (Yes‐Associated Protein)‐(TEA Domain Transcription Factor) signaling, which drives tumor growth and therapy resistance. However, VP is limited by poor solubility, low tumor selectivity, and systemic toxicity. Researchers from the group of Song et al. [207], have developed these biocompatible NPs to improve the targeting of UM tumors, the efficacy of PDT, and to leverage YAP inhibition for synergistic therapy. HANP were prepared from hyaluronic acid conjugated with hydrophobic 5β‐cholanic acid. VP was encapsulated using high‐pressure homogenization. HANP/VP were 200 nm in diameter, stable in physiological buffers for 7 days, and with a 90% loading efficiency. HANP were (hyaluronidase)‐responsive enzyme, with 76% VP released in 30 h under acidic tumor‐like conditions. HANP/VP produced more ROS than free VP in cells as measured with 2′,7′‐dichlorodihydrofluorescein diacetate (DCFH‐DA) assay. Cytotoxicity was tested in human UM (92‐1) and murine UM (B16F10) cell lines with or without laser irradiation (690 nm). HANP/VP killed more tumor cells after laser irradiation. At 2 µM VP with laser, around 81%–90% cell death was observed with HANP/VP versus around 61%–68% with free VP. Without light, HANP/VP still suppressed growth by inhibiting YAP. In vivo tumor targeting was assessed with fluorescence imaging in a B16F10 mouse model. Tumors were obtained by injecting tumor cells into the right and left flanks of mice. HANP/VP accumulated three times more in tumors than free VP (via CD44 binding and EPR effect) that accumulated mainly in liver, kidney, and lungs (risking toxicity). HANP/VP alone, in the absence of light, slowed tumor growth through YAP inhibition. However, when combined with laser irradiation, HANP/VP significantly inhibited tumor growth, achieving a threefold greater reduction compared with the control group. Free VP was less effective than HANP/VP with or without light activation. Histological and molecular analysis showed a reduced proliferation (Ki67 was downregulated) and increased apoptosis (Caspase‐3 was upregulated) in cells treated with HANP/VP combined with laser. YAP expression was also markedly downregulated (79% of downregulation with HANP/VP with laser) and an increased infiltration of CD8^+^ T cells and M1‐type macrophages (CD68+) were observed in HANP/VP with laser group which led to an improved anti‐tumor immunity. No significant systemic toxicity or weight loss in treated mice were observed.

**FIGURE 4:**
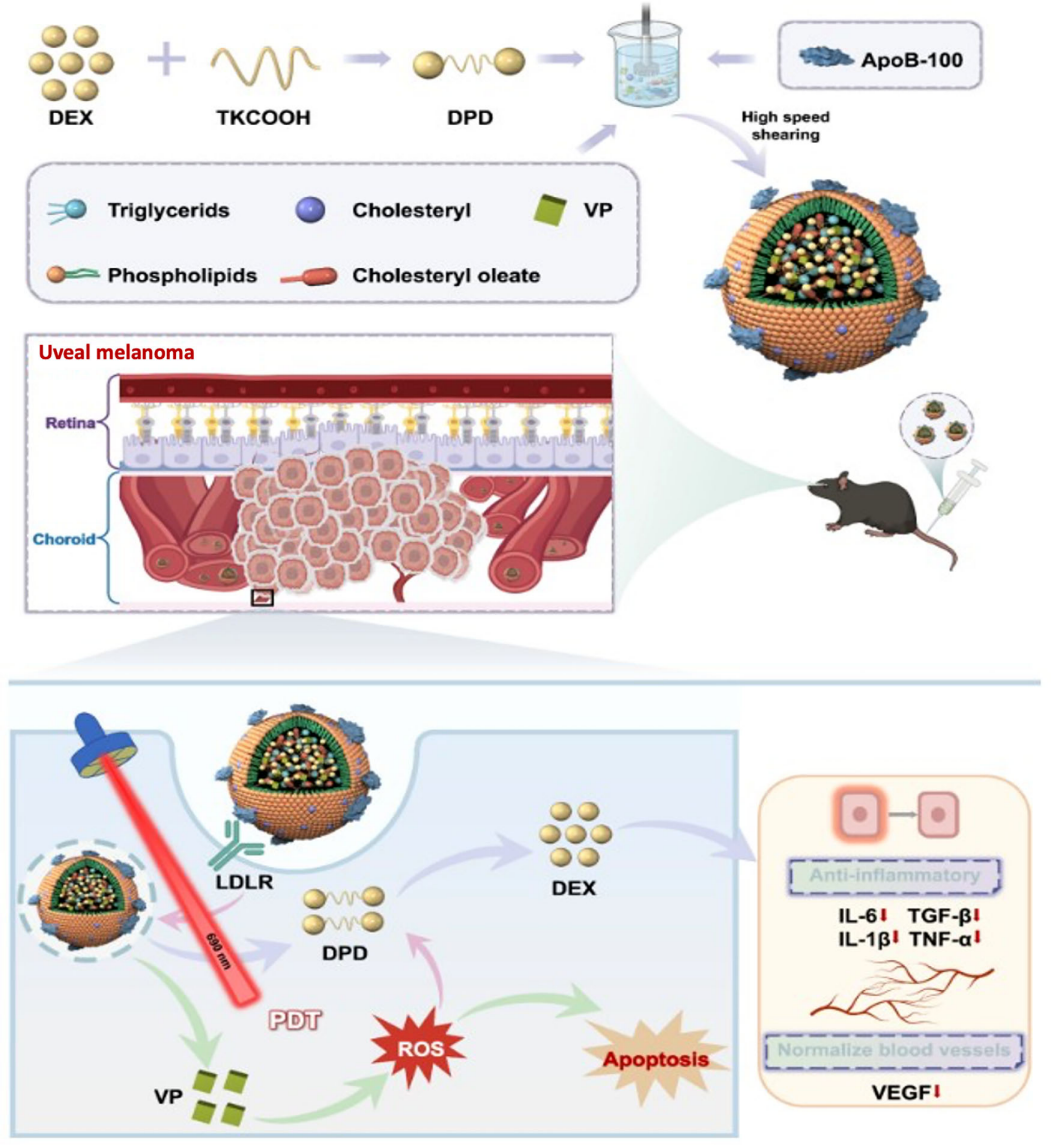
A schematic of a drug‐loaded low‐density lipoprotein NP delivery system for tumor therapy. LD‐DPVP NPs are targeted to tumor cells via ApoB‐100. Laser‐induced ROS generation cleaved thioketal bonds triggering DEX release, which normalizes blood vessels and reduces inflammation at the tumor site. Reproduced with permission [210]. Copyright 2025, Elsevier.

PDT using VP is promising, but its efficacy is restricted by abnormal tumor vasculature. Dexamethasone (DEX) can normalize tumor blood vessels and improve NP penetration. It can reduce inflammation and angiogenesis but has systemic side effects if used in high doses. Bi et al. [208] therefore developed biomimetic low‐density lipoprotein NPs (LD‐DPVP NPs) of 120 nm size, with high drug‐loading efficiency (90%). They were stable in serum and during storage and were nonhemolytic. LD‐DPVP NPs encapsulated VP photosensitizer and a ROS‐responsive DEX prodrug (DPD). LD‐DPVP NPs mimic natural LDL to achieve active targeting via Apolipoprotein B‐100 (ApoB‐100)/low‐density lipoprotein receptor (LDLR). Indeed, tumor cells (B16F10) showed much higher uptake than normal retinal cells (ARPE‐19). Uptake was blocked when ApoB competed for LDLR, confirming active targeting. The system was designed to release drugs only when stimulated with NIR (690 nm) laser light. Indeed, VP generated abundant ROS under light, triggering fast and controlled DEX release through the cleavage of the ROS‐sensitive linker in DPD. In vitro, a strong cytotoxicity against melanoma cells under light (apoptosis up to 85%) and a reduction of B16F10 cell migration and invasion was observed. Furthermore, LD‐DPVP NPs with laser inhibited tube formation of human umbilical vein endothelial cells (HUVECs) showing the anti‐angiogenic effect of the system. Mice treated with LD‐DPVP NPs (intravenous injection) maintained body weight and showed no organ toxicity, unlike free DEX+VP solution. NPs were biocompatible with minimal systemic toxicity. LD‐DPVP NPs (intravenous injection) + laser showed the strongest tumor suppression, reduced angiogenesis, improved vascular maturity, and lowered VEGF expression in an orthotopic model of UM. The system was safe: no structural damage to cornea or retina under laser was observed. However, eye‐drop delivery showed poor efficacy because of a low ocular bioavailability (Figure 4).

**FIGURE 5:**
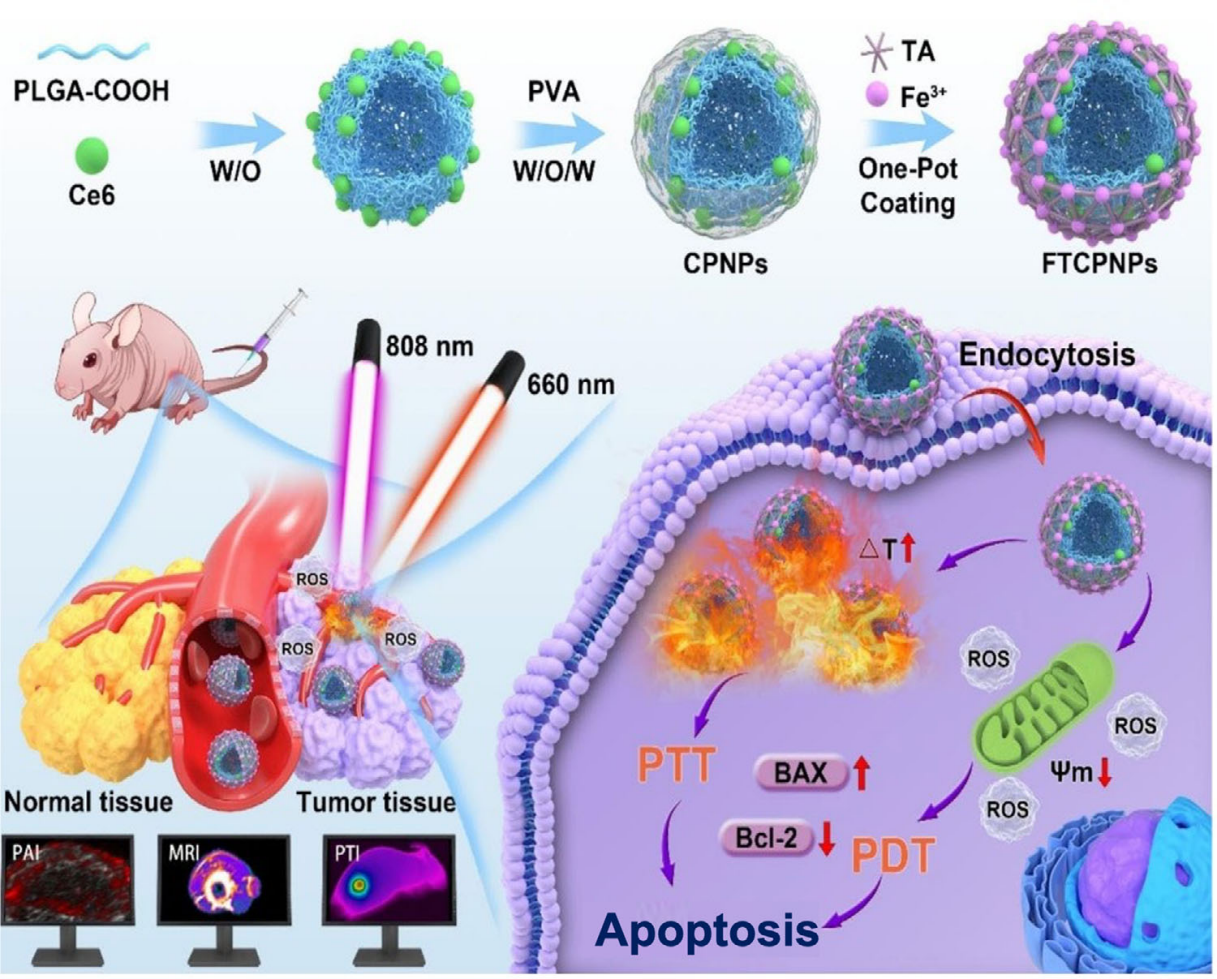
FTCPNPs synthesis and combination effects of PTT/PDT against UM tumors. Following tumor accumulation, FTCPNPs enable MRI and PAI imaging and, under 808 nm and 660 nm irradiation, induce heat and ROS generation, respectively. The combined treatment promotes mitochondrial dysfunction, Bax upregulation, Bcl‐2 downregulation, and apoptosis, resulting in greater antitumor efficacy than either PTT or PDT alone. Reproduced with permission [211]. Copyright 2023, Springer.

An additional explored therapeutic approach described in the literature aimed at developing multifunctional NPs capable of combining PTT and PDT for UM. The first example was reported by the group of Huang et al. [209], who synthesized FeIII‐TA/PLGA/Ce6 NPs (FTCPNPs). The NPs enable dual‐modal magnetic resonance imaging (MRI) and photoacoustic imaging (PAI) to guide therapy. PLGA encapsulated the photosensitizer Ce6. Fe(III)‐tannic acid (Fe(III)‐TA) coating enhanced photothermal conversion and provided MRI/PAI contrast. The NPs showed a uniform spherical core–shell structure, high Ce6‐loading efficiency, stability, and paramagnetic properties. FTCPNPs exhibited excellent biocompatibility in normal cell lines (ARPE‐19). Upon 808 and 660 nm NIR irradiation, FTCPNPs generated heat (PTT) and ROS (PDT), respectively. FTCPNPs with both lasers triggered mitochondrial dysfunction, membrane depolarization, and apoptosis in UM cells (C918). FTCPNPs combined with dual‐laser irradiation induced significantly higher tumor cell death compared to PTT or PDT alone. RNA‐sequencing revealed upregulation of apoptosis‐related genes, with downregulation of anti‐apoptotic proteins (e.g., Bcl‐2) and upregulation of pro‐apoptotic Bax (Figure 5). MRI and PAI demonstrated that FTCPNPs accumulated in tumor tissue, peaking at 24 h post‐injection in mice with UM xenografts. Fluorescent labeling confirmed biodistribution with preferential accumulation in tumors, and metabolism occurring mainly via the liver and spleen. Dual‐mode treatment eradicated tumors without recurrence over 14 days. Minimal systemic toxicity was observed; mice maintained stable body weight and showed no organ damage. Routine blood tests and histological analyses confirmed no major toxic side effects up to 28 days post‐treatment.

**FIGURE 6:**
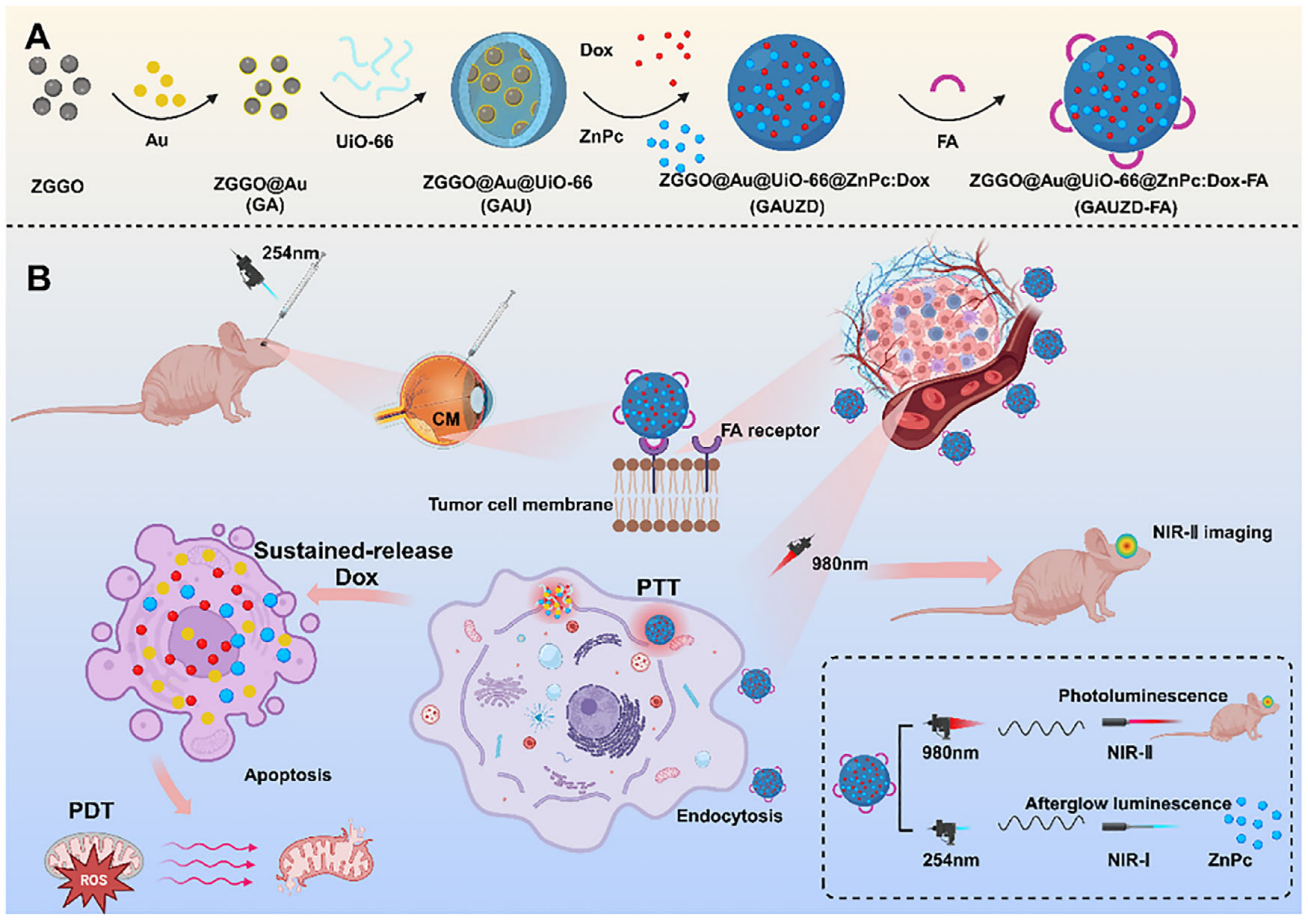
Schematic illustration of (A) construction of multifunctional hybrid nano long afterglow material GAUZD‐FA for the treatment of CM and (B) the NIR‐II imaging and triple‐synergistic therapy of choroidal melanoma. Following FA‐mediated tumor targeting, GAUZD‐FA enables NIR‐II imaging and combines PTT, PDT, and sustained doxorubicin release. The combined treatment enhances cellular uptake, induces mitochondrial damage and apoptosis, and achieves superior tumor inhibition compared with non‐targeted or single‐modality treatments. Reproduced with permission [214]. Copyright 2025, Elsevier.

A further study on the PDT/PTT synergistic effect reports the development of a multifunctional long afterglow hybrid nanoplatform (GAUZD‐FA) for the diagnosis and treatment of choroidal melanoma (CM) for the first time. The team of Yang et al. [212] designed a multifunctional nanoplatform: ZGGO@Au@UiO‐66@ZnPc:Dox‐FA (GAUZD‐FA) composed of Core (ZGGO): Zn_1.3_ Ga_1.4_ Ge_0.3_ O_4_:Cr_0.004_ Yb_0.04_ Er_0.004_ persistent luminescent NPs, Au NPs: photothermal agent, UiO‐66 (metal–organic framework (MOF)): carrier for sustained doxorubicin (Dox) release, ZnPc: phthalocyanine for PDT, Dox: chemotherapy drug, Folic acid (FA): active tumor‐targeting ligand. GAUZD‐FA possesses imaging properties with Strong NIR‐II luminescence for deep tissue penetration, and low background at 980 nm excitation. NIR‐I afterglow luminescence avoids continuous irradiation after excitation at 254 nm. GAUZD‐FA possesses good intraocular retention for real‐time monitoring. GAUZD‐FA allowed tritherapy. PTT: Au enabled localized hyperthermia under 980 nm laser. PDT: ZnPc activated by ZGGO afterglow/upconversion generated ^1^O_2_. Chemotherapy: sustained release of Dox within TME with high loading efficiency (72.5%) and drug content (15.1%) was observed. The monitoring of controlled release, led to 96.7% release over 48 h, with prolonged effect up to 120 h. FA modification enhanced uptake by tumor cells (OCM‐1), sparing normal cells (ARPE‐19). GAUZD‐FA showed good biocompatibility in vitro (low toxicity without Dox). Synergistic effect of PDT + PTT + Dox markedly reduced CM cell survival to ∼29% in vitro. FA targeting enhanced efficacy compared to nontargeted versions. JC‐1 probe and flow cytometry confirmed strong mitochondrial damage and apoptosis induction. In vivo biosafety was confirmed by liver/kidney function tests and histology. In vivo treatment with an orthotopic model of CM allowed the demonstration of the better targeting efficiency of GAUZD‐FA by NIR‐II imaging. Intravitreal GAUZD‐FA injection + 980 nm irradiation inhibited tumor growth significantly. GAUZD‐FA were irradiated with a 254 nm laser before the injection to trigger afterglow luminescence and PDT. GAUZD‐FA achieved safe, effective tumor ablation with minimal damage to surrounding tissue, and enabled single injection, multiple treatments via long afterglow, and sustained drug release (Figure 6).

**FIGURE 7:**
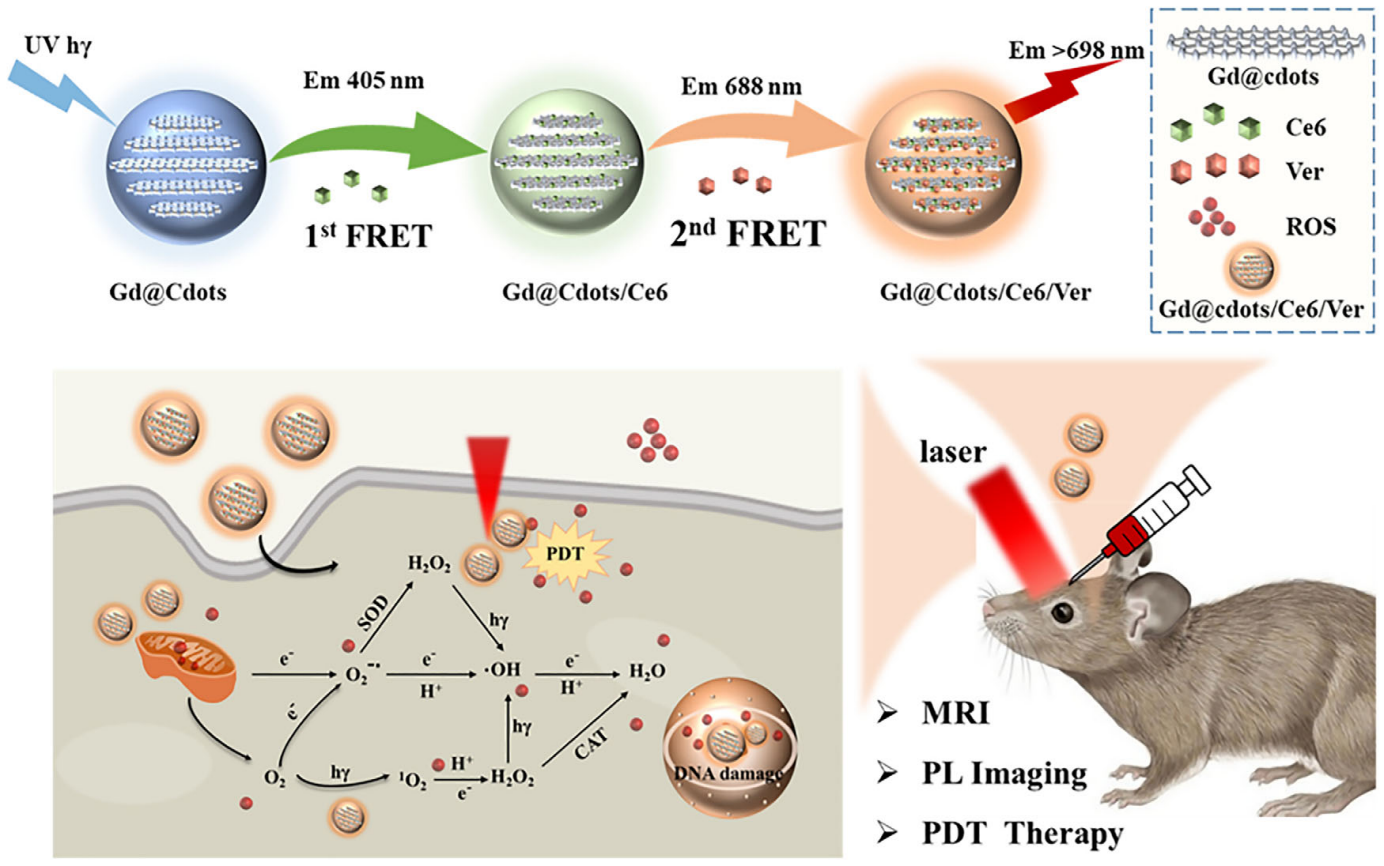
A cascade FRET photosensitizer that yielding greater ^1^O_2_ production to enhance PDT of UM. Gadolinium (Gd)‐doped carbon dots (Cdots) conjugated with Ce6 and Ver yielded photosensitizing cascade FRET NPs (D/A1/A2 = Gd@Cdots/Ce6/Ver). These NPs generate ^1^O_2_, which is easily quenched and then cascaded produce amounts of ·OH at an acidic microenvironment in photodynamic therapy (PDT) of UM in orthotopic tumor‐bearing mice. Reproduced with permission [213]. Copyright 2022, Elsevier.

In addition, in a notable alternative approach, Ruan et al. present the development and evaluation of a novel cascade Förster resonance energy transfer (FRET)‐based photosensitizer NP, designed to enhance PDT effectiveness for treating UM [211]. They designed a Gd@Cdots/Ce6/Ver photosensitizer, which consists of: gadolinium‐doped carbon dots (Gd@Cdots) as energy donors, Ce6 and Verteporfin (Ver in this study) as energy acceptors. The NP generated ^1^O_2_ and other ROS through: a cascade FRET process (Gd@Cdots → Ce6 → Ver), direct FRET from Gd@Cdots to Ce6 or Ver, ROS production upon UV light activation, inducing tumor cell death. Gd@Cdots/Ce6/Ver showed higher ROS production, particularly ^1^O_2_, ·OH, and O_2_^−^·, than other photosensitizers tested in vitro at a low concentration of 50 µg/mL. Enhanced apoptosis, mitochondrial damage, and inhibition of cell proliferation and migration in human UM cell lines (MUM‐2B, OCM‐1) were observed. Intracellular localization showed strong nuclear targeting. Gd@Cdots/Ce6/Ver were then evaluated in subcutaneous mouse models. After intravenous injection, the NPs were imaged with fluorescence and MRI techniques. PDT using Gd@Cdots/Ce6/Ver significantly reduced tumor size and weight. An orthotopic model expressing luciferase was also used. Bioluminescent signals in the ocular tumors and tumor weight both indicated a lower tumor growth rate with the Gd@Cdots/Ce6/Ver. No significant toxicity was observed in major organs or blood panels. Although activated by UV light, the cascade FRET photosensitizer presented here enhances ^1^O_2_ production, improving PDT efficacy in UM, and illustrating a promising concept that could be applied to NIR light activation (Figure 7).

**FIGURE 8:**
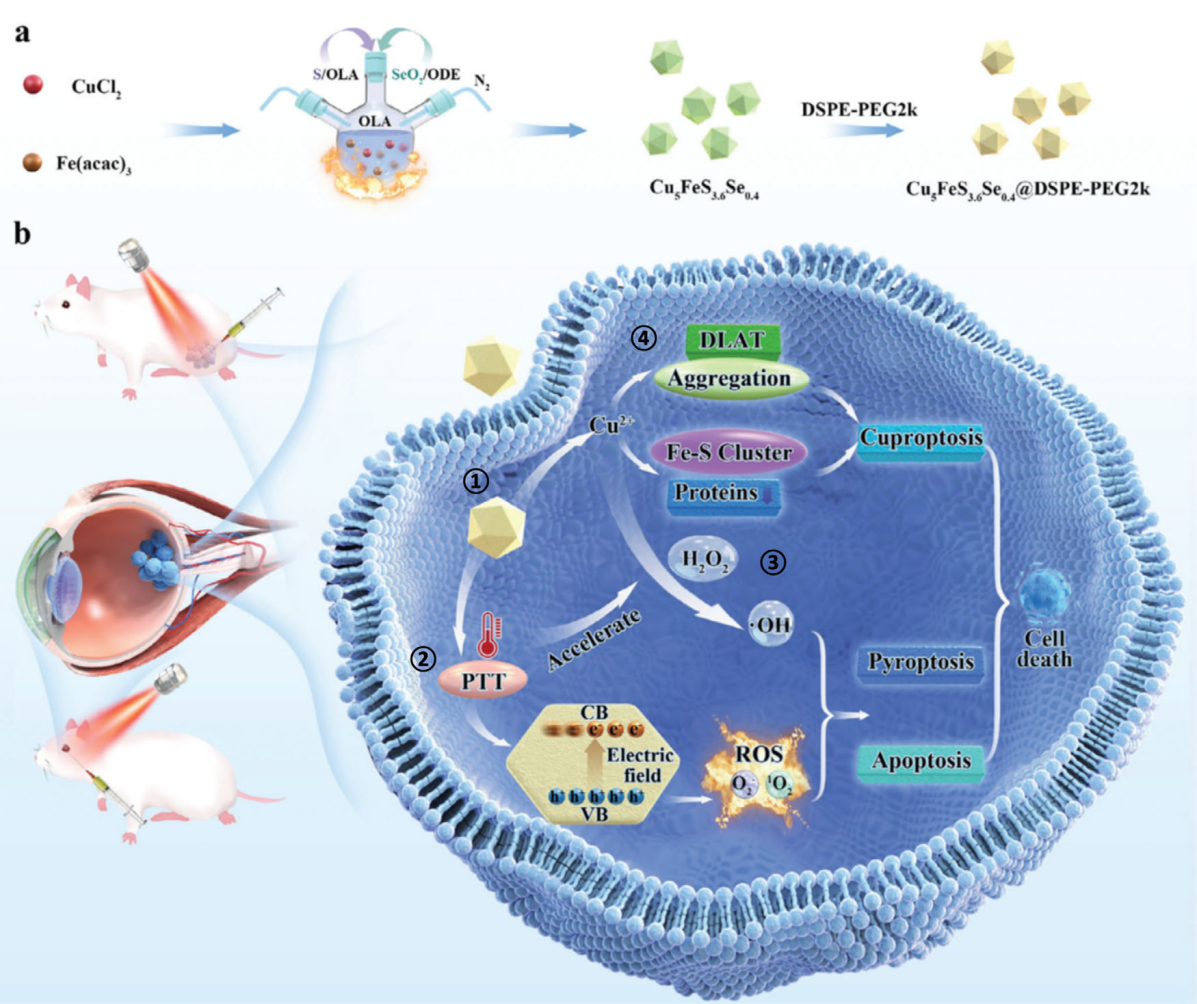
a) Schematic illustration of the Cu_5_FeS_3.6_Se_0.4_ NPs construction and b) their therapeutic mechanism. 1) Under NIR light irradiation, Cu_5_FeS_3.6_Se_0.4_ NPs initiate PTT. 2) Hyperthermia generates thermoelectric potentials in Cu_5_FeS_3.6_Se_0.4_ NPs, facilitating the production of ROS. 3) Cu_2+_ released from Cu_5_FeS_3.6_Se_0.4_ NPs evokes CDT by catalyzing H_2_O_2_ to produce OH, which is accelerated by hyperthermia. 4) Cu_5_FeS_3.6_Se_0.4_ NPs cause DLAT aggregation and downregulate the expression of Fe‐S clusters, leading to cuproptosis. The synergistic combination of photothermal killing, thermoelectric effects, and CDT induces cellular cuproptosis, apoptosis, and pyroptosis, eventually leading to cell death. Reproduced with permission [212]. Copyright 2025, Wiley.

Finally, a recent noteworthy strategy [210] involves exploiting multifunctional thermoelectric nanocatalysts based on energy conversion, particularly Cu_5_FeS_3.6_Se_0.4_ NPs, to stimulate the generation of ROS and chemodynamic therapy (CDT), that selectively kills tumor cells via pyroptosis and cuproptosis. Photothermal conversion under a NIR laser (1064 nm) induces localized hyperthermia. The thermoelectric effect converts thermal gradients into electrical potentials, thereby stimulating the production of ROS. CDT, through the release of Cu^+^ and Fe, catalyzes Fenton‐type reactions with intratumoral H_2_O_2_, generating toxic hydroxyl radicals (·OH). Glutathione depletion prevents the neutralization of ROS, exacerbating oxidative stress. Surface modification with a modified DSPE‐PEG_2_k phospholipid improves stability and biocompatibility. The Cu_5_FeS_3.6_Se_0.4_ NPs, with a diameter of 270 nm and a cubic crystal structure, exhibit strong NIR absorption and high photothermal stability (conversion efficiency: 34.1%). They exhibit increased voltage and conductivity under the influence of heat and a laser; selenium doping improves charge carrier mobility. The mechanisms of antitumor action were investigated. PTT directly destroys tumor cells. The production of thermoelectric ROS, via the electron flow induced by hyperthermia, increases oxidative stress. The production of chemodynamic ROS, through the catalysis of H_2_O_2_ to ·OH, is accelerated by heat. Three cell death mechanisms are involved: apoptosis, cuproptosis, triggered by the release of Cu^2^^+^, which leads to the disorganization of Fe‐S clusters and the aggregation of DLAT and ROS‐induced pyroptosis that activates caspase‐8, leading to gasdermin C cleavage and pore formation in the cell membrane. NPs in the presence of tumor cells induced a potent cytotoxic effect under NIR irradiation. The combination of NPs + H_2_O_2_ + laser proved most effective (only ∼22.5% of tumor cells survived). A high rate of apoptosis (50.4%) was confirmed by flow cytometry. A subcutaneous tumor model (mouse) was also studied. Tumor temperature reached over 54 °C under 1064 nm laser irradiation after intratumoral injection of NPs. Approximately 86.6% inhibition of tumor growth was observed after 13 days. The survival rate was 80% at day 40, compared to rapid mortality in the control group. Cu_5_FeS_3.6_Se_0.4_ NPs exhibit minimal systemic toxicity; no organ damage or weight loss was observed. An orthotopic ocular tumor model was studied. The combination of NPs and laser prevented intraocular tumor expansion. Ocular structure and retinal function were confirmed by OCT and electroretinography (ERG). Marker of proliferation Ki‐67 (Ki67)/ Terminal deoxynucleotidyl transferase dUTP Nick‐End Labeling (TUNEL) staining confirmed decreased proliferation and increased apoptosis. Increased NOD‐, LRR‐, and Pyrin Domain‐Containing Protein 3 (NLRP3) and Dihydrolipoamide S‐Acetyltransferase (DLAT) expression confirmed the involvement of pyroptosis and cuproptosis (Figure 8).

The nanosystems presented in this section underscore the potential of nanotechnology to integrate multiple therapeutic modalities within a single platform. Across studies, these NPs exhibit a favorable safety profile with minimal systemic toxicity. They facilitate synergistic interactions among treatment options, thereby offering promising avenues for improvements in the management of UM. Notably, the combination of immunotherapy with PDT appears particularly effective for preventing metastasis and tumor recurrence. Activation of NPs within the NIR confers substantial advantages for both imaging and therapy, owing to its enhanced tissue penetration and compatibility with light‐based ocular treatments, thus holding considerable potential for targeting intraocular tumors. Mechanisms of action, advantages, and limitations for each of the described nanosystems are summarized in Table 5.

**TABLE 5:** Current clinical and preclinical NP‐assisted phototherapy strategies investigated for UM.

Phototherapy strategy	Representative nanosystem	Nanoplatform category	Mechanism of action	Advantages	Limitations	Refs.
**PDT**	AU‐011 (IR700‐conjugated VLPs, Phase III)	VLPs	HSPG‐targeted binding followed by 690 nm photoactivation, inducing ROS generation, localized membrane disruption, immunogenic cell death, and antitumor immune activation	High tumor selectivity, preservation of healthy ocular tissues, low systemic toxicity; potential induction of antitumor immunity, first clinically advanced nanophoto‐therapy in UM	Requires local light exposure and HSPG expression, currently mainly applicable to local disease	[233, 234, 235]
**PDT + immunotherapy**	FIC‐PDT + Ripasudil + anti‐PD‐L1	Polymeric nanophotosensitizers	Ce6‐mediated PDT induces ICD and DAMP release (CRT, HMGB1, ATP, HSP70), ROCK inhibition promotes APC phagocytosis, while anti‐PD‐L1 restores cytotoxic T‐cell activity	Converts immunologically “cold” tumors into “hot” tumors, enhances CD8^+^ T‐cell infiltration, suppresses primary and metastatic tumors, promotes systemic antitumor immunity	Complex multi‐agent treatment, dependent on immune competence and efficient light delivery	[224]
**PDT + cGAS‐STING activation**	NP^PDT^‐56MESS	ROS‐responsive polymeric NPs	PDT‐triggered ROS release platinum‐based 56MESS, inducing DNA damage and activation of the cGAS‐STING pathway, leading to dendritic‐cell maturation and immune‐memory formation	Long‐term antitumor immunity, prevention of recurrence and metastasis, tumor‐selective activation, reduced systemic toxicity	Complex synthesis and formulation, currently limited to preclinical validation	[225]
**PDT + Vascular normalization + ICB**	Combo‐NP (PDT‐NP + Len‐NP)	Polymeric NPs	PDT‐induced ICD combined with ROS‐triggered lenvatinib release, vascular normalization, hypoxia reduction, enhanced CTL infiltration, and improved response to anti‐PD‐L1 therapy	Overcomes hypoxia‐associated PDT resistance; enhances immune infiltration, induces abscopal effects, suppresses metastatic progression	Multifunctional formulation with challenging manufacturing and clinical translation	[206]
**PDT + Molecular targeting (YAP inhibition)**	HANP/VP NPs	Hyaluronic acid NPs	CD44‐targeted VP delivery combines ROS‐mediated PDT with inhibition of the YAP‐TEAD signaling pathway	Enhanced tumor accumulation, increased ROS generation; dual antitumor mechanism, improved immune‐cell infiltration, low systemic toxicity	Dependent on VP activation and CD44 expression, preclinical stage	[207]
**PDT + Anti‐angiogenic therapy**	LD‐DPVP NPs	Biomimetic LDL NPs	LDLR‐targeted delivery of VP and dexamethasone, PDT‐induced ROS trigger‐controlled drug release, vascular normalization, and anti‐angiogenic effects	Active tumor targeting, controlled drug release, reduced VEGF expression; improved vascular maturity, favorable safety profile	Limited efficacy via topical administration, intravenous delivery required	[208]
**Combined PDT/PTT**	FTCPNPs	FeIII‐TA/PLGA hybrid NPs	Ce6‐mediated PDT (660 nm) combined with FeIII‐TA‐mediated PTT (808 nm); MRI and photoacoustic imaging‐guided therapy	Synergistic ROS and thermal damage, image‐guided treatment; complete tumor eradication without recurrence, good biocompatibility	Requires dual‐laser irradiation, relatively complex formulation	[209]
**PDT/PTT + Chemotherapy + Imaging**	GAUZD‐FA	MOF‐based hybrid NPs	Au‐mediated PTT, ZnPc‐mediated PDT, sustained doxorubicin release, folate targeting, and NIR‐II imaging	Triple‐synergy therapy; prolonged drug release, real‐time monitoring, high tumor selectivity, multimodal therapeutic action	Highly complex architecture, difficult scale‐up and regulatory approval	[212]
**Enhanced PDT (FRET‐amplified PDT)**	Gd@Cdots/Ce6/Ver NPs	Carbon‐dot nanoplatforms	Cascade FRET amplifies singlet oxygen and ROS generation, enhancing apoptosis and inhibiting tumor progression	Increased PDT efficiency, MRI capability, enhanced ROS production, improved apoptosis induction	UV‐light activation, limited tissue penetration, proof‐of‐concept stage	[211]
**PTT + CDT**	Cu_5_FeS_3.6_Se_0.4_ NPs	Thermoelectric nanocatalysts	NIR‐triggered PTT combined with thermoelectric ROS generation, Fenton‐like CDT, glutathione depletion, cuproptosis, pyroptosis, and apoptosis	Multiple complementary cell‐death pathways, strong tumor inhibition, preserved retinal structure and function, minimal systemic toxicity	Complex mechanism and formulation, early‐stage development with limited validation	[210]

### 4.4 Key Challenges, Safety Concerns, and Translational Considerations for Um

The translational relevance of NP‐based phototherapy strategies in UM should be evaluated according to their capacity to address major biological and therapeutic challenges inherent to UM, including effective intraocular delivery, optical limitations associated with tumor pigmentation, preservation of visual function, and potential impact on metastatic progression. While the multiple nanoplatforms described in this section demonstrate promising antitumor activity, the level of evidence and translational maturity remains heterogeneous across the currently available approaches.

First, a central consideration concerns the relevance of preclinical models used for therapeutic evaluation. Several studies rely on surrogate melanoma systems, particularly B16F10, owing to their robustness, reproducibility, rapid growth kinetics, and compatibility with immunocompetent hosts (C57BL/6 mice). Although such models do not reproduce the molecular landscape of UM, including alterations in GNAQ, GNA11, BAP1, SF3B1, or EIF1AX, they remain useful for investigating fundamental phototherapeutic parameters. In particular, the strong pigmentation of B16F10 tumors provides an experimentally relevant platform for assessing light attenuation and melanin‐mediated quenching of ROS, which constitute important barriers in UM phototherapy. Accordingly, these systems remain valuable for initial validation of photophysical performance and therapeutic mechanisms, including enhancement of local photosensitizer accumulation using nanoformulated VP [207], or evaluation of biomimetic and stimuli‐responsive NPs [208]. Compared with cutaneous melanoma, relatively few immunocompetent murine models of UM are available for investigating tumor progression and antitumor immune responses. Although more representative systems, including orthotopic intraocular models, have been developed, they remain less standardized, more costly, and technically demanding. Orthotopic models require delicate intraocular implantation procedures in a highly confined anatomical structure, with risks of ocular damage and challenges in delivering precise irradiation to posterior ocular tissues. Human UM cell lines (e.g., 92.1, MP38, MP46, and Mel270) are convenient human models, because they are simple, cheap, and accessible but remain extremely limited mostly because of their lack of physiological relevance (as 2D models). Consequently, proof‐of‐concept studies of novel photosensitizers or NP‐based platforms are often initially performed in simpler experimental systems before addressing the biological and technical complexity of intraocular models. Emerging human relevant models (or NAMs for new approach methodologies) could open translational approaches and serve as future preclinical testing platforms using patient‐derived tumor organoids, recreating blood flow with vasculature‐on‐chip, and simulating BOB for NPs penetration [238].

Second, achieving safe and effective intraocular biodistribution within the uveal tract represents a key translational consideration. Delivery efficiency depends on both the administration route and NP characteristics, including size, composition, surface properties, and biodegradation profile (as described in Table 3). Multiple administration routes have been explored for ocular NP delivery, including systemic, intravitreal, subconjunctival/periocular, and suprachoroidal approaches. Among these, the suprachoroidal route appears particularly promising for uveal targeting because it enhances delivery to the choroid and adjacent posterior tissues while reducing anterior and systemic exposure. Its clinical relevance is supported by the development of belzupacap sarotalocan (AU‐011), a photoactivatable virus‐like particle administered suprachoroidally for small choroidal melanoma, which has demonstrated encouraging safety and tumor‐control outcomes [233, 234]. In contrast, efficient and selective targeting of the ciliary body and iris remains less extensively characterized, as current delivery strategies are primarily optimized for posterior ocular tissues. Therefore, extending NP‐based approaches to these structures will require dedicated studies on biodistribution, ocular toxicity, pharmacokinetics, and functional safety before clinical translation.

Third, the compatibility between activation wavelength and the optical properties of ocular tissues is a critical determinant of therapeutic performance. Most phototherapeutic systems operate within the red or clinically validated NIR‐I window (∼650–900 nm), where ocular PDT is already established, as illustrated clinically by the 689 nm activation of belzupacap sarotalocan (AU‐011) for choroidal melanoma [233, 234]. However, high melanin content in the retinal pigment epithelium, choroid, and tumor tissue strongly attenuates light and competes with photosensitizers for photon absorption, reducing treatment efficacy in highly pigmented tumors. Emerging strategies, including NIR‐II‐responsive platforms [212], engineered excitation strategies such as cascade FRET mechanisms [211], and two‐photon excitation approaches, may overcome these limitations by enabling more efficient depth‐resolved activation within deeply pigmented tissues, although their clinical translation requires further validation regarding retinal safety, dosimetry, and tissue‐specific accessibility within the uveal tract.

Fourth, potential preservation of visual function also is an important consideration when comparing phototherapeutic approaches with conventional radiotherapy. PDT may theoretically provide improved visual preservation because of its localized tumor destruction and reduced exposure of adjacent retinal and optic structures. Clinical studies using VP‐based PDT have demonstrated favorable ocular tolerability [34, 113, 187]. Similarly, NP‐mediated phototherapies, including clinically advanced systems such as AU‐011 [233, 234], have been designed to enhance lesion‐selective treatment through improved photosensitizer delivery and localized activation. Severe vision loss is reported in 43% of patients after brachytherapy and up to 80% following PBT for tumors near critical visual structures [239, 240, 241]. In contrast, phase II studies of belzupacap sarotalocan (AU‐011) reported visual acuity preservation in approximately 90% of treated patients. Although these approaches suggest a favorable safety profile and potential functional advantages for targeted photoactivatable therapies, direct evidence demonstrating superior long‐term visual outcomes compared with established modalities such as brachytherapy or PBT remains limited.

Finally, the photodynamic and NP‐based phototherapy approaches discussed in this review are primarily designed for local control of the primary tumor, with reported benefits mainly related to localized tumor eradication and preservation of surrounding ocular structures. However, whether these strategies influence metastatic risk remains largely unresolved. In UM, metastatic dissemination may occur years before clinical diagnosis, and improved local tumor control does not necessarily translate into enhanced survival outcomes. Although experimental studies suggest that phototherapy may induce broader biological effects, extending beyond the treated lesion, including immune checkpoint sensitization following immunogenic tumor clearance [224], and cGAS–STING‐mediated systemic immune activation [225], current evidence remains predominantly preclinical and insufficient to demonstrate clinically meaningful effects on metastatic progression.

Overall, translational maturity is highest for (i) clinically validated or NIR photosensitizer delivery systems [233, 234] and (ii) optimized photophysical activation strategies such as FRET‐based or NIR‐II‐responsive platforms [211, 212]. In contrast, immune reprogramming, vascular normalization, and programmed cell death modulation represent promising but still evolving extensions of NP‐mediated phototherapy, requiring further validation in UM‐specific ocular models and clinically relevant metastatic settings.

Critical translational assessment of NP‐based phototherapeutic approaches described in Section 4.3 is summarized in Tables 6 and 7.

**TABLE 6:** Critical assessment for translational relevance to UM of experimental models used in NP‐based phototherapy studies.

Representative nanosystem	Experimental model(s) used	Model classification	Relevance for UM	Refs.
AU‐011 **(IR700‐conjugated VLPs, Phase III)**	Human UM 92.1 cells (primary UM; GNAQ‐mutant) in vitro; subcutaneous xenografts in nude mice; orthotopic rabbit intraocular xenograft model	Authentic orthotopic UM model + UM‐specific xenograft model	High—Reproduces intraocular anatomy and local treatment conditions; stronger translational relevance than most preclinical studies. Combines authentic human UM cells with an orthotopic intraocular model. Subcutaneous xenografts do not reproduce ocular immune privilege, choroidal vascularization, or metastatic tropism. Rabbit xenografts improve anatomical relevance but still lack complete human UM microenvironment complexity.	[233]
AU‐011 **(IR700‐conjugated VLPs, Phase III)**	Human subjects with primary indeterminate lesions or small choroidal melanoma. Human clinical trial	Clinical/ authentic human UM	Very high—Directly reflects human UM biology, treatment response, and functional outcomes. Highest translational relevance. Treatment evaluated directly in patients with small choroidal melanoma. Experimental model limitations no longer apply.	[234]
AU‐011 **(IR700‐conjugated VLPs, Phase III)**	Primary UM cell lines (92.1, Mel270, Mel285, MP38, MP46) and metastatic UM lines (OMM1, OMM2.3, OMM2.5, MM28, MM66)	UM‐specific in vitro model	Moderate—high—Strong biological relevance. Both primary and metastatic UM cell lines evaluated, including BAP1‐associated phenotypes. Lacks intraocular architecture, immune privilege, or metastatic dissemination (in vitro systems).	[235]
**FIC‐PDT + Ripasudil +** **anti‐PD‐L1**	B16F10 melanoma intraocular model	Surrogate intraocular melanoma model	Moderate—Preserves ocular localization and immune interactions but lacks UM‐specific genetics (GNAQ/GNA11, BAP1, chromosome 3 abnormalities) Significant limitation for UM translation.	[224]
**NP^PDT^‐56MESS**	Human UM 92.1 cells in vitro; B16‐F10 syngeneic melanoma model for in vivo immune studies	UM‐specific in vitro + surrogate melanoma model	Moderate—low—Useful for immune mechanisms but does not reproduce ocular immune privilege or authentic UM metastatic behavior.	[225]
**Combo‐NP (PDT‐NP + Len‐NP)**	Human UM C918 and MUM‐2B cells with murine xenograft studies	UM‐specific xenograft model	Moderate—Reproduces selected UM tumor characteristics but lacks native ocular environment and immune context.	[206]
**HANP/VP NPs**	Human UM 92.1 and MEL270 cells; subcutaneous xenograft mice	UM‐specific xenograft model	Moderate—Preserves UM molecular background but not ocular anatomy nor immune privilege.	[207]
**LD‐DPVP NPs**	Human UM 92.1 and C918 cells with xenograft validation	UM‐specific xenograft model	Moderate—Relevant for evaluating tumor targeting and therapeutic efficacy, with limited physiological representation.	[208]
**FTCPNPs**	Human UM C918 and MUM‐2B cells; subcutaneous xenograft models	UM‐specific xenograft model	Moderate—Suitable for assessing imaging‐guided phototherapy but does not reproduce ocular constraints.	[209]
**GAUZD‐FA**	Choroidal melanoma model using C918‐derived intraocular/xenograft systems	Authentic orthotopic UM model	Moderate—high—Anatomically relevant choroidal models. Reproduces ocular localization but limited immune representation.	[212]
**Gd@Cdots/Ce6/Ver NPs**	Human UM C918 cells with murine xenograft validation	UM‐specific xenograft model	Moderate—Useful for photophysical optimization but lacks UM microenvironment.	[211]
**Cu_5_FeS_3.6_Se_0.4_ NPs**	Human UM 92.1 and MUM‐2B cells with xenograft models	UM‐specific xenograft model	Moderate—Mechanistically relevant but does not reproduce ocular or metastatic complexity.	[210]

**TABLE 7:** Translational potential of NP‐based phototherapies for UM.

Represen‐tative nanosystem	Delivery feasibility to choroid/ciliary body/iris	Optical compatibility (wavelength/ pigmentation)	Potential for vision preservation vs radiotherapy	Impact on metastatic disease	Most translatable component	Refs.
AU‐011 **(IR700‐conjugated VLPs, Phase III)**	Designed for localized intraocular administration, appears compatible with posterior segment delivery.	NIR activation provides improved tissue penetration.	May potentially reduce collateral retinal injury and preserve visual function compared with radiotherapy.	Primarily focused on local tumor eradication, metastatic benefit not demonstrated	Targeted photosensitizer delivery, clinically adaptable laser protocols	[233]
AU‐011 **(IR700‐conjugated VLPs, Phase III)**	Specifically designed for intraocular clinical administration.	Optical parameters optimized for ocular application	Preservation of visual function	Focused on local disease control	Treatment protocol, localized phototherapeutic delivery	[234]
AU‐011 **(IR700‐conjugated VLPs, Phase III)**	Delivery characteristics derived from the AU‐011 platform but not directly evaluated.	Similar optical considerations to AU‐011	Cannot be determined from in vitro observations.	Immunological relevance, metastatic benefit remains speculative	Immunogenic cell death and immune activation mechanisms	[235]
**FIC‐PDT + Ripasudil +** **anti‐PD‐L1**	Additional validation is required regarding intraocular delivery safety.	PDT activation may be limited by melanin‐associated optical attenuation.	Retinal phototoxicity remains insufficiently characterized.	Demonstrated systemic immune responses and inhibition of metastatic dissemination	Combination of immunogenic PDT with ICB	[224]
**NP^PDT^‐56MESS**	Long‐term ocular biodistribution and safety require additional characterization.	NP‐mediated enhancement may improve photodynamic efficiency despite pigmentation constraints.	Potentially favorable due to localized activity. Functional vision outcomes undefined.	Suggests systemic anti‐tumor immune responses	cGAS‐STING‐mediated immunogenic activation	[225]
**Combo‐NP (PDT‐NP + Len‐NP)**	Ocular safety of NIR‐II NP systems incompletely characterized.	NIR‐II wavelengths provide superior penetration and reduced scattering through pigmented tissues.	Reduced collateral damage may theoretically improve functional preservation.	Demonstrated anti‐metastatic activity in experimental settings	Vascular normalization combined with checkpoint blockade	[206]
**HANP/VP NPs**	Additional validation of ocular retention and posterior‐segment delivery is needed.	VP benefits from established clinical photodynamic properties.	Lower irradiation requirements may potentially reduce ocular damage.	Primarily directed toward local disease control	Nanoformulated photosensitizer delivery combined with molecular targeted therapy	[207]
**LD‐DPVP NPs**	Biomimetic nanoplatform safety within intraocular tissues requires additional investigation.	Depends on the excitation mechanism employed	Functional ocular preservation remains undetermined	Predominantly local therapeutic effects	Precision‐targeted NP delivery systems	[208]
**FTCPNPs**	Long‐term ocular safety data remain limited.	Image‐guided cooperative phototherapy may improve treatment precision and energy deposition.	Precision‐guided treatment may reduce collateral retinal exposure.	Mainly directed toward local tumor control	Image‐guided phototherapeutic platforms	[209]
**GAUZD‐FA**	Biodistribution and ocular toxicity require additional investigation.	NIR‐II activation is advantageous for deep penetration through highly pigmented ocular tissues.	Spatially restricted activation may improve preservation of visual function.	Primarily local tumor effects	NIR‐II imaging‐guided multimodal treatment strategy	[210]
**Gd@Cdots/Ce6/Ver NPs**	Ocular retention and safety remain insufficiently characterized.	Enhanced energy transfer may permit lower irradiation doses	Reduced energy exposure may theoretically reduce retinal injury	Predominantly local therapeutic activity	Photosensitizer engineering and photodynamic optimization	[211]
**Cu_5_FeS_3.6_Se_0.4_ NPs**	Ocular nanocatalyst safety requires additional investigation.	Compatibility depends on excitation conditions and thermal effects.	Potential effects on visual function remain unclear.	Mainly local therapeutic outcomes reported	Exploitation of cuproptosis and pyroptosis mechanisms in nanotherapy	[210]

Although most NP‐mediated approaches have demonstrated promising preclinical efficacy, translation into UM remains limited, and further studies addressing ocular delivery feasibility, biodistribution, long‐term safety and vision preservation, and clinical scalability will be necessary. Currently, the most clinically translatable aspects of NP‐assisted PDT in UM are the optimization of photosensitizer delivery and illumination parameters, as both are built upon already established ophthalmic PDT principles and existing laser technologies. NPs may enhance photosensitizer biodistribution, stability, intratumoral accumulation, and local retention while minimizing off‐target exposure, as illustrated by clinically advanced systems such as AU‐011 [233, 234]. Likewise, optimized optical activation strategies, particularly in the NIR‐II range, may enhance therapeutic precision [210]. In contrast, strategies involving immune modulation, vascular reprogramming, or combination with immune ICIs remain largely preclinical and have not yet demonstrated clinical benefit in mUM. Furthermore, the clinical translation of NP systems themselves remains challenging, requiring extensive evaluation of biodistribution, pharmacokinetics, ocular safety, intraocular behavior, manufacturing reproducibility, and regulatory compliance before routine clinical implementation.

## 5 Outlook/Near‐Term Research Priorities toward Clinical Translation of NP Phototherapy for UM

Despite the rapid emergence of increasingly sophisticated NP‐based phototherapeutic platforms, the transition from experimental proof‐of‐concept to a clinically viable therapy for UM will likely depend less on the generation of increasingly sophisticated nanosystems alone and more on addressing a series of practical, biological, and disease‐specific constraints.

Unlike many solid tumors, therapeutic success in UM is not defined solely by local tumor eradication. The principal objective is simultaneously to achieve durable local control while preserving ocular integrity and visual function. Consequently, future therapeutic strategies must maintain a delicate balance between antitumor efficacy and protection of highly specialized ocular structures including the retina, retinal pigment epithelium, macula, optic nerve, and choroid. This consideration is particularly important because current eye‐conserving therapies, including brachytherapy and PBT, already provide satisfactory local control but remain associated with substantial visual morbidity, especially for tumors located near the fovea or optic disc. Therefore, demonstration of superior tumor destruction alone may be insufficient and future studies will need to establish meaningful functional benefits in terms of visual preservation.

A major short‐term priority concerns the establishment of safe, reproducible, and clinically feasible delivery strategies. The ocular environment presents unique anatomical barriers, which considerably restrict drug penetration and reduce the efficiency of systemic administration. While local administration strategies may improve exposure at the tumor site, their translation requires a detailed understanding of NP biodistribution, tissue retention, biodegradation, clearance pathways, inflammatory responses, and long‐term ocular toxicity. Beyond demonstrating anti‐tumor activity, future studies must establish whether sufficient tumor accumulation can be achieved while minimizing exposure of adjacent ocular tissues.

In parallel, the success of nanomedicine in UM will depend not only on biological performance but also on scalability of production, reproducibility between batches, storage stability, sterilization procedures, and compliance with regulatory requirements for clinical‐grade formulations. Similar considerations apply to light delivery itself, as therapeutic efficacy remains strongly dependent on irradiation parameters including wavelength, fluence, irradiance, treatment duration, and treatment scheduling. The large variability currently observed across preclinical phototherapy studies complicates comparisons between systems and limits the establishment of standardized treatment protocols compatible with routine ophthalmic practice. Future research should also progressively move beyond simplified proof‐of‐concept systems toward models that more faithfully reproduce the anatomical, optical, and biological characteristics of human UM. Such models will be important for evaluating treatment behavior under conditions that better reflect intraocular delivery constraints, tumor pigmentation, ocular immune regulation, and metastatic dissemination.

Beyond these translational considerations, optimization of light–tissue interactions itself represents an important direction for future development. Future progress will depend not only on improving NP design but also on optimizing light delivery, exploiting the unique optical properties of the eye. Conventional phototherapies rely predominantly on single‐photon excitation, which is limited by relatively poor tissue penetration and significant photon attenuation by melanin, acting as a major optical barrier. Among emerging alternatives, two‐photon PDT (2P‐PDT) has attracted considerable interest because it employs the simultaneous absorption of two NIR photons, enabling greater tissue penetration while restricting excitation to the focal volume. This spatial confinement provides superior three‐dimensional precision, minimizes phototoxicity to adjacent ocular structures, and offers improved spatiotemporal control of photosensitizer activation, making it particularly attractive for treating tumors located close to critical visual structures [242].

Recent experimental evidence further suggests that pulsed two‐photon femtosecond laser excitation may fundamentally modify the interaction between light, melanin, and photosensitizers. In a murine model of UM, Pires et al. demonstrated superior therapeutic efficacy of femtosecond 2P‐PDT compared with conventional single‐photon PDT, particularly in pigmented tumors [243]. Interestingly, the efficacy of PDT no longer depends solely on the intrinsic properties of the photosensitizer, but also on the cellular environment, particularly the presence of melanin. This phenomenon suggests that the two‐photon cross‐section alone is an insufficient criterion for predicting therapeutic efficacy under real biological conditions. Melanin therefore acts as a natural amplifier, making pigmented melanomas particularly sensitive to 2P‐PDT, while for UM with little or no pigmentation, the use of photosensitizers with a high two‐photon cross‐section remains essential. Interestingly, photodynamic performance did not correlate solely with the intrinsic two‐photon absorption properties of the photosensitizer. Instead, melanin appeared to actively participate in energy transfer, behaving as a multiphoton antenna capable of transferring absorbed energy toward photosensitizers, through mechanisms including short‐range nonradiative energy transfer processes resembling FRET or indirect radiative transfer through melanin‐associated fluorescence emission. These observations challenge the traditional view of melanin as merely an optical barrier and suggest that, under appropriate excitation conditions, tumor pigmentation could become a therapeutic advantage rather than a limitation. These findings are particularly intriguing given the availability of clinical two‐photon fluorescence scanning laser ophthalmoscope and imaging technologies, potentially facilitating future translational development [244].

Although most current phototherapeutic systems operate within the NIR‐I region (700–900 nm), the emergence of NIR‐II technologies offers significant optical advantages. Compared with NIR‐I, NIR‐II wavelengths exhibit reduced photon scattering and lower tissue autofluorescence, resulting in improved signal‐to‐noise ratio, enhanced spatial resolution, and greater penetration depth. In addition, the higher maximum permissible exposure associated with NIR‐II wavelengths may allow the delivery of greater light doses without compromising safety, representing an important advantage for both PDT and PTT [245, 246, 247, 248, 249].

Finally, future progress will likely rely increasingly on multifunctional and multimodal theranostic platforms integrating imaging guidance, phototherapy, and controlled therapeutic delivery. By combining optimized excitation wavelengths with image‐guided treatment and controlled therapeutic release, these systems offer improved tumor selectivity, real‐time treatment monitoring, and precise spatiotemporal control of therapy. Nevertheless, near‐term priorities should remain focused on demonstrating reproducible safety, clinically feasible delivery procedures, standardized irradiation protocols, and measurable improvements in visual outcomes compared with current eye‐conserving therapies. Ultimately, the transition of NP phototherapy toward a realistic UM treatment paradigm will likely emerge from the convergence of technological innovation with clinically actionable and biologically relevant translational strategies.

## 6 Conclusion

This review explores the current landscape of UM management, with a particular focus on laser‐ and photo‐induced therapies, suitable for ophthalmology in general, and their recent evolution through nanotechnology. While conventional treatments such as radiotherapy and surgery remain effective for local tumor control, they are associated with significant side effects, including ocular toxicity, anatomical constraints, and a limited impact on metastatic progression, which remains the primary cause of mortality. Laser‐based therapies, including TTT and PDT, provide minimally invasive and spatially controlled alternatives. However, their efficacy is constrained by limited living tissue penetration, tumor pigmentation, and variable clinical responses, often leading to incomplete tumor eradication or recurrence. In this context, NP‐assisted phototherapies represent a major advancement. By improving tumor targeting, enhancing photosensitizer delivery, and enabling controlled activation, nanoplatforms significantly increase therapeutic precision while reducing systemic toxicity. They allow synergistic multimodal approaches, combining PDT, TTT, and systemic treatments such as immunotherapy or chemotherapy. Although still largely at the preclinical stage in UM, these approaches show strong potential for improving both local and systemic disease control.

Looking forward, clinical translation will require overcoming key challenges: optimizing NP biodistribution in ocular and hepatic tissues, ensuring reproducible safety profiles, and designing light delivery systems compatible with deep or pigmented lesions. In particular, the use of NIR‐II (1000–1700 nm) light offers greater tissue penetration, reduced scattering, and minimized off‐target phototoxicity, enabling more effective activation of photosensitizers in thick or heavily pigmented tumors. Future studies should focus on standardized preclinical models of UM, scalable manufacturing of multifunctional nanoplatforms, and early‐phase clinical trials with rigorous imaging and functional endpoints. In addition, combinatorial approaches that integrate immunomodulation, TME normalization, and NIR‐II‐triggered therapies could enhance systemic control of micrometastases. Together, these strategies provide a realistic roadmap for advancing NP‐based phototherapies from preclinical promise toward safe and effective clinical use in UM.

## Author Contributions

**Emilie Lambert**: conceptualization, investigation, writing – original draft, methodology. **Christophe Nguyen**: methodology, investigation, validation, writing – original draft. **Sean Edward Dunn**: investigation, writing – original draft, methodology. **Magali Gary‐Bobo**: conceptualization, writing – original draft, methodology, validation. **Jérôme Lacombe**: investigation, funding acquisition, writing – original draft, validation, project administration. **Justin Moser**: investigation, writing – original draft, validation. **Frédéric Zenhausern**: investigation, funding acquisition, writing – original draft, validation, supervision, project administration. **Jean-Olivier Durand**: investigation, writing – original draft, methodology, validation. **Frédérique Cunin**: conceptualization, investigation, funding acquisition, writing – original draft, methodology, validation, supervision, project administration.

## Conflicts of Interest

The authors declare no conflicts of interest.

## Data Availability

The data that support the findings of this study are available from the corresponding author upon reasonable request.
